# Global Trends in Diabetic Foot Research (2004–2023): A Bibliometric Study Based on the Scopus Database

**DOI:** 10.3390/ijerph22040463

**Published:** 2025-03-21

**Authors:** Yolanda Fuentes-Peñaranda, Alma Labarta-González-Vallarino, Elena Arroyo-Bello, Marina Gómez de Quero Córdoba

**Affiliations:** 1 Department of Nursing, Facultad de Enfermería, Fisioterapia y Podología, Universidad Complutense de Madrid, 28040 Madrid, Spain; 2 Independent Researcher, 28220 Madrid, Spain; allabart@yahoo.com; 3 Escuela de Enfermería Fundación Jiménez Díaz, Universidad Autónoma de Madrid, 28040 Madrid, Spain; elena.arroyo@inv.uam.es; 4 Instituto de Investigación Sanitaria, Hospital Fundación Jiménez Díaz, Universidad Autónoma de Madrid, 28040 Madrid, Spain; 5 Facultat Infermeria, Universitat Rovira i Virgili, 43002 Tarragona, Spain; marina.gomezdequero@urv.cat

**Keywords:** diabetic foot, bibliometric analysis, research trends, Scopus

## Abstract

Diabetic foot is one of the leading complications of diabetes mellitus that affects millions of people around the world and involves the presence of ulcers, infections, tissue destruction, and loss of sensation and can even lead to limb amputation. This research explores trends in diabetic foot global research through a bibliometric analysis of publications indexed in Scopus in the period 2004–2023. A total of 7136 documents were analysed using Excel, Python, Biblioshiny, and VOSviewer. Scientific production has multiplied by a factor of 6.6 from the first to the last year analysed. Armstrong D.G. is the most productive and cited author. China is the most productive country, and the United States is the most cited. The most productive journal is the *International Journal of Lower Extremity Wounds*, and the most cited journal is *Diabetes Care*. Research on diabetic foot is mainly focused on the complications of diabetes mellitus; the treatment and healing of wounds; infections; and epidemiology and patient care. Infections and antibiotic treatment are emerging topics, while deep learning and machine learning are among the niche topics in this area of knowledge. The present study allows us to identify current trends and future directions of research in diabetic foot.

## 1. Introduction

Diabetes mellitus is a very important public health problem [1,2,3], being one of the leading causes of death and disability worldwide and affecting all people regardless of their country, age group, or sex [2]. The Global Burden of Diseases, Injuries, and Risk Factors Study (GBD) estimates that there will be 1.31 billion people living with diabetes by 2050 [2], and the IDF Diabetes Atlas estimates that diabetes-related healthcare expenditures globally will reach the amount of USD 1.054 trillion by 2045 [3].

The most prevalent diabetes is type 2 diabetes, which reaches more than 96% of cases worldwide, and most of the people suffering from it are over 65 years of age [2]. In addition to major cardiovascular, kidney, and eye diseases, people with diabetes are at high risk for a serious complication known as diabetic foot [1]. This is defined as “Disease of the foot of a person with current or previously diagnosed diabetes mellitus that includes one or more of the following: peripheral neuropathy, peripheral artery disease, infection, ulcer(s), neuro-osteoarthropathy, gangrene, or amputation” [4].

Diabetic foot is one of the most feared and complex complications, as it is associated with very high rates of lower limb amputation and increased morbidity and mortality [5,6]. Approximately 18.6 million people worldwide suffer diabetic foot ulcers each year, and these ulcers precede 80% of lower limb amputations in people diagnosed with diabetes [7]. It is estimated that up to a quarter of patients with diabetes are at risk of developing diabetic foot [5] and between 19% and 34% are at risk of developing foot ulcers over the course of their lifetime [1]. Approximately 50% of diabetic foot ulcers become infected and 20% of people with diabetic foot ulcers will undergo lower limb amputation [7]. It is estimated that the five-year amputation rate is 10% and the five-year mortality rate is 40%. Unfortunately, even when a cure is achieved, 40% of patients experience a recurrence within one year [5]. All these alarming statistics justify the need for active treatment to prevent and treat diabetes and its complications, including diabetic foot, to optimise quality of life and to reduce the economic burden on health care systems [8].

The high prevalence of diabetic foot and its fearsome consequences for the patient and society have generated multitude of research, and there is a need to know the current state of emerging issues, which are the already-investigated areas as well as those others in which it would be necessary to further promote research. We are living in an era marked by the exponential growth of scientific production, and bibliometric analyses have become an indispensable tool for understanding the dynamics of the different fields of research [9].

Donthu et al. [10] note that researchers use bibliometric analyses to discover emerging trends, to evaluate the performance of articles and journals, to analyse collaboration patterns and research components, and to examine the intellectual structure of a particular area in the existing literature. Bibliometric studies have gained popularity in recent years as a rigorous method that allows large volumes of scientific data to be explored and analysed with an interdisciplinary methodology [10,11]. Bibliometric studies have been developed in all subject areas and are widely used in the fields of medicine and health research [9].

In the scientific literature, we can find bibliometric studies that deal with general aspects of diabetes mellitus [8,12,13,14,15,16,17] and also bibliometric studies that address aspects related to diabetic foot [18,19,20,21,22,23,24,25,26,27,28,29,30,31,32,33] in which different topics are covered such as trends in global research on diabetic foot [18,25,32,33], research at the regional level [21,22,24], nursing care [27,31], diagnostic techniques [30], treatment [28,29], ulcers in diabetic foot [19,20,26,27,29], or risk factors [23], among other topics. This research is carried out by authors from different geographical backgrounds, among which China predominates and stands out [18,19,20,25,26,27,28,29,31,33].

The main objective of this study is to determine the characteristics and trends of global research on the diabetic foot by analysing publications indexed in the Scopus database in the last two decades (2004–2023). The specific objectives proposed are

1. To determine the general characteristics of the published documents;
2. To analyse the production and citation of the most relevant authors, countries, and journals, as well as the collaboration networks generated between authors and countries;
3. To identify the most cited documents and bibliographic references most used by researchers in the field of diabetic foot;
4. To analyse keywords and to identify motor, basic, emerging, and niche topics within this area of knowledge.

All of this will provide very valuable information to health professionals and researchers interested in the field of diabetic foot, showing its development and future lines of research.

## 2. Materials and Methods

Comprehensive and transparent bibliometric reporting is essential to improve the reliability, interpretability, and value of research findings [34]. There are numerous publications that intend to publicise bibliometric methodology [9,10,11,34,35,36,37,38,39,40,41,42,43], showing techniques and good practices to improve the quality and clarity of this type of research; despite this, currently there is no clear guideline for bibliometric reporting recognised by the international scientific community [34].

The present study is carried out following the BIBLIO methodological guide recently proposed by Montazeri et al. [40] for the performance of transparent and complete bibliometric studies available in the EQUATOR network (Enhancing the QUAlity and Transparency Of health Research) [44], an international initiative that aims to disseminate publication guidelines and monitor their use to improve the quality of scientific communication. The compliance in this study with the recommendations given by BIBLIO is shown in the Supplementary Materials to this publication, in Table S1.

The usual techniques for developing a bibliometric study cover two basic fronts: performance analysis (which accounts for the contributions of the different components of the research) and scientific mapping (which focuses on the relationships among these components) [11,35,36,38,41,42,43], which we will address in our study.

### 2.1. Design and Information Sources

An observational study of a bibliometric nature and of international scope is carried out based on the traditional laws of bibliometrics [45]. The sources of information used for this study are the Scopus database [46] (Elsevier, Amsterdam, The Netherlands) for data export and three databases—Scimago Journal and Country Rank (SJR) [47], Journal Citation Reports (JCR) [48], and Information Matrix for Journal Analysis (MIAR) [49]—to analyse the characteristics and main bibliometric indicators of the journals.

Scopus is a highly reliable multidisciplinary bibliographic database containing abstracts and citations, with more than 94 million records currently [46]. It is recognised within the academic community as one of the main databases for bibliometric studies along with the Web of Science, and both contain a wide variety of internationally renowned scientific journals of high impact and quality [50]. Scopus has a greater documentary coverage, a greater geographical coverage at the European level than Web of Science, and identifies authors’ profiles in a more specific way, so we have decided to use it as a reference source for this study.

SJR is based on the information contained in Scopus and provides numerous bibliometric indicators on journals and countries. JCR is based on the information collected from the citations received by the indexed articles of the main collection of the Web of Science, and provides bibliographic information of the journals, as well as the bibliometric indicators of the most internationally recognised journals. And MIAR is based on more than 100 sources, corresponding to journal repertoires and international bibliographic databases, and provides useful information for the identification of journals and the analysis of their dissemination.

### 2.2. Search Strategy

To carry out the bibliographic search, the MeSH term “diabetic foot” [51] was used, and it was executed using quotation marks (“”) in the title field to ensure a better fit to the subject of the data. Only articles and review documents were selected, as these are the ones that mainly receive citations, and any other types of records retrieved were excluded. There was no language restriction. The search was later on limited to the period between 1 January 2004 and 31 December 2023, spanning the last two decades. The year 2024 was not included as the search was conducted on 28 July 2024. The strategy executed was as follows:

TITLE (“diabetic foot”) AND PUBYEAR > 2003 AND PUBYEAR < 2024 AND (EXCLUDE (DOCTYPE, “cp”) OR EXCLUDE (DOCTYPE, “ch”) OR EXCLUDE (DOCTYPE, “le”) OR EXCLUDE (DOCTYPE, “no”) OR EXCLUDE (DOCTYPE, “sh”) OR EXCLUDE (DOCTYPE, “ed”) OR EXCLUDE (DOCTYPE, “er”) OR EXCLUDE (DOCTYPE, “bk”) OR EXCLUDE (DOCTYPE, “tb”) OR EXCLUDE (DOCTYPE, “cr”) OR EXCLUDE (DOCTYPE, “dp”) OR EXCLUDE (DOCTYPE, “cb”).

A total of 7176 records were obtained and exported with all the fields available in Scopus (citation information, bibliographic information, abstract data and keywords, funding details, and other information) in CSV format.

### 2.3. Data Collection and Extraction

All these data were imported into Microsoft Excel 365 to examine their validity through sorting and filtering of the different fields and checking duplicates of document titles. Cleaning and purification of the data were carried out. Two researchers (E.A.B. and Y.F.P.) worked independently on these processes to ensure their quality and replicability. With the control of duplicates through the conditional format of Microsoft Excel, 192 documents with possible duplications were identified. The coincidences between the different fields of the duplicated records were checked, and when required, the full texts were also checked. Any doubts that arose between the researchers were resolved through sharing and debate. When a consensus was not reached, differences were resolved by a third researcher (A.L.G.V.). After this review, 40 records were removed from the sample. The final sample to be analysed was 7136 records.

**Figure 1:**
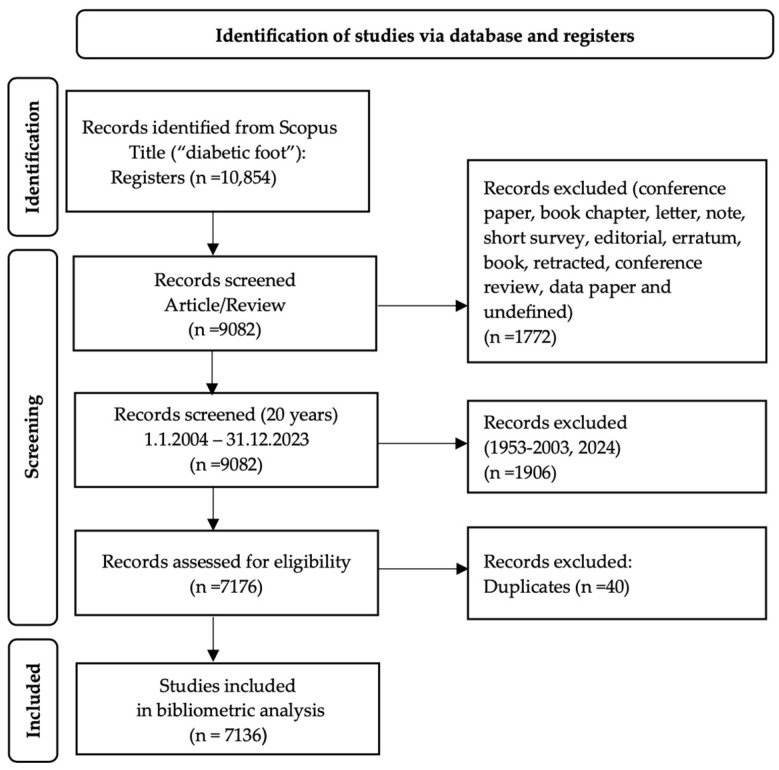
Flowchart of data filtering process and publications excluded.

The selection of the records made is shown in Figure 1.

### 2.4. Data Analysis

For the data analysis to be valid, it was necessary to carry out different review processes in which data were cleaned and normalised both automatically and manually. All data were retrieved from the Scopus database and imported into different software tools for statistical and visual analysis. Microsoft Excel 365, Python v.3.11, the Bibliometrix R package v.4.3.0, through its Biblioshiny application, and VOSviewer v.1.9.20 were used.

We used the Python programming language to check records and eliminate duplicates within the same field, to normalise data, and generate CSV files. For the identification and normalisation of authors, the fields *Author Full Names* and *Author(s) IDs* were used, the different author signatures were unified with the same code, and the signature variations and the position of the authors within the documents were identified. For keyword normalisation, the *Author Keywords* (DEs) and *Index Keywords* (IDs) fields were used. DEs are the terms chosen by the authors themselves to describe the content of their research and are an effective tool for investigating the structure of knowledge in any scientific field [39]. IDs are terms chosen by Scopus and are standardised according to vocabularies derived from thesauri that Elsevier owns or is licensed to use. Duplicate terms within the same field were checked and automatically eliminated.

Microsoft Excel was used to manually clean and normalise data, for its graphical representation and to perform the descriptive analysis of the sample. In manual debugging and normalisation, synonymous terms were unified in the fields that referred to keywords, journals, countries, and authors. Once manually debugged, they were imported back into Python to replace the final terms in the parent file and generate a CSV file readable for the rest of the applications used.

For bibliometric mapping we used VOSviewer, software developed by Van Eck and Waltman [52] as it works very well with large data sets; allows the connection between items through links of cocitation, co-occurrence, citations, and bibliographic coupling; and offers three types of visual maps: network, overlay, and density visualisation [28,52]. With this software, we have generated maps of collaborative networks between authors and countries and thematic maps of keyword co-occurrences that show the relationships between terms [11]. In order not to lose information that could be relevant, both the authors’ keywords (DE) and those provided by the Scopus logarithm (ID) were analysed. We also want to reflect that, given the high number of components involved in these analyses (authors, countries, and keywords) to generate more visual and understandable maps, only a limited number of these components—the most relevant—were selected. In the case of authors, only large producers with 10 or more publications and a maximum of 10 signatures per article were selected; in the case of countries, only those that submitted at least 20 publications were selected; and in the case of keywords, only those that had at least 15 repetitions were selected.

For a deeper understanding of the subject, we used Bibliometrix, an open-source program, through its Biblioshiny application developed by Aria and Cuccurullo [53] to obtain some specific bibliometric indicators provided by this application. These are as follows: 1. local H-index, 2. number of publications, 3. number of citations, 4. citation averages, 5. frequency of publications divided into authors, and 6. degree of collaboration between countries taking into account the corresponding author. It has also been used for downloading data related to countries, the most cited documents, and the most consulted references, as well as for graphical representation of production over time by authors and countries, word clouds, and thematic maps.

To show a visualisation of the figures that is clearer and more focused on the topics of interest, regarding the keywords, the main term “diabetic foot” was hidden, and terms were unified and eliminated through the creation of different thesauri, both for the VOSviewer application and for Biblioshiny. The terms eliminated with these thesauri were those referring to very general concepts (“adult”, “male”, “female”, “human”, “nonhuman”, “article”) or to the identification of different methodological designs, among others, and were excluded due to their lack of contribution to the graphic representation. The terms related to different types of major and minor amputations were unified under the term “amputation”, and the terms “diabetic foot syndrome” and “diabetic foot disease” were unified under “diabetic foot”. Each application provided unique advantages, and together, they offered a comprehensive analysis of the research on diabetic foot.

### 2.5. Ethical Statement

The present study uses bibliographic records exported from the Scopus database and bibliometric data provided by SJR, JCR, and MIAR and does not involve human subjects; therefore, it is not subject to the ethical review requirements for biomedical research.

## 3. Results

Table 1 presents the summary of the characteristics of the 7136 documents analysed on diabetic foot between 2004 and 2023.

### 3.1. Overall Analysis of the Documents

#### 3.1.1. Types of Documents and Language

In the 7136 documents analysed, a total of 5956 articles and 1180 reviews were counted; 41.17% of the documents are available in open access, of which 85.70% are articles and 14.30% are reviews.

There is wide linguistic coverage in the documents indexed by Scopus. In our sample, we found publications in 28 different languages. Most of the papers are published in English (82.54%; *n* = 5890), followed in frequency by Chinese (5.17%; *n* = 369), German (2.56%; *n* = 183), Russian (2.51%; *n* = 179), Spanish (2.02%; *n* = 144), and French (1.32%; *n* = 94). The remaining 3.88% are documents published in 22 other languages. The total distribution of publication languages of the documents is shown in Supplementary Materials in Table S2.

#### 3.1.2. Distribution of Documents and Citations by Year

In the field of diabetic foot, a mean and standard deviation of 356.8 ± 199 documents were published per year, with the mode being 244. The minimum was 118, in 2004, and the maximum was 779 documents, in 2023, indicating that it has multiplied its annual production 6.6 times from the first to the last year analysed. A trend of exponential growth is observed in compliance with Price’s Law, with production doubling after 10 years. The first decade (2004–2013) added a total of 1599 documents, and in the second (2014–2023), 4357 documents were added, which multiplied production by 2.7 times.

**Figure 2:**
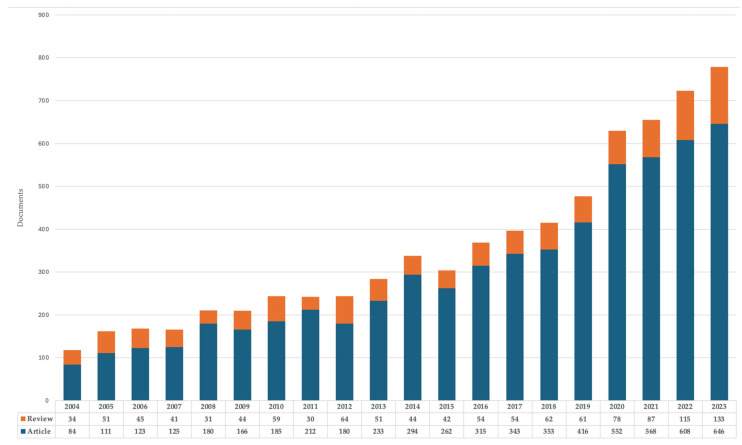
Annual growth of the typologies of papers on diabetic foot in Scopus from 2004 to 2023.

When analysing by document type, we observe that article-type documents multiplied their growth 7.7 times and revision-type documents multiplied their growth 3.9 times in the 20-year period analysed. The distribution by year and type of document is shown in Figure 2.

**Figure 3:**
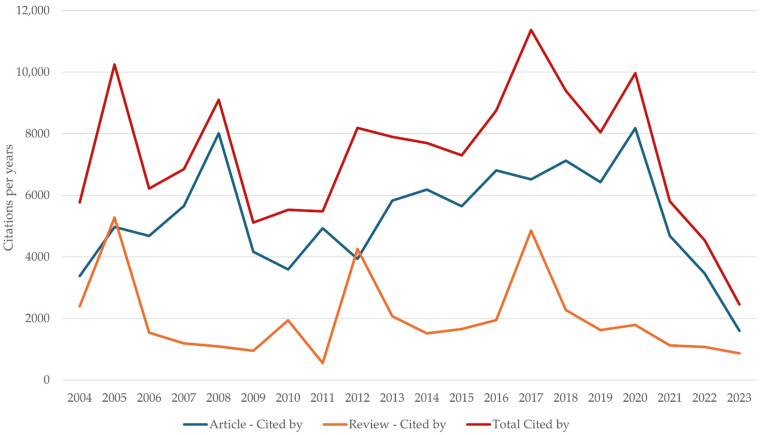
Distribution of citations received according to document type over time.

These documents have received a total of 145,688 citations, with the mean and standard deviation being 7284.4 ± 2203.96 citations per year. The distribution of citations received by year and type of document is shown in Figure 3.

### 3.2. Author Analysis

#### 3.2.1. Analysis of Authors’ Production and Citations

In the 7136 documents analysed, a total of 23,898 different authors were counted, with 37,604 signatures or occurrences. There were 32 papers in which Scopus did not index any authors, and the maximum number of authors signing a paper was 41. The average number of signatures was 5.27 ± 3.40, with the mode being four authors per document. The collaboration rate between authors is 98.45%; the remaining 1.55% (582) correspond to authors who published alone.

**Figure 4:**
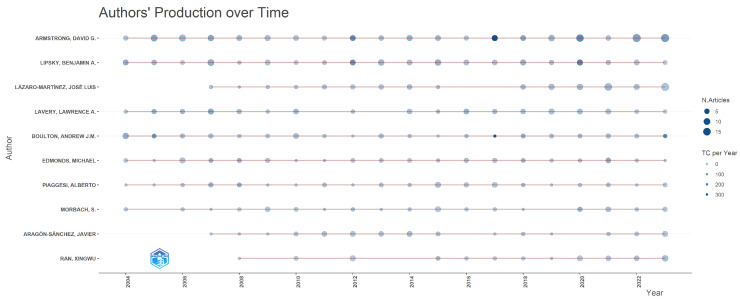
Top 10 most productive authors over time in the field of diabetic foot (2004–2023).

In the sample analysed, Lotka’s Law or the law of author productivity is fulfilled, which says that most scientific knowledge comes from a small group of very productive authors. We counted 237 (0.99%) large producers (authors who have published 10 or more documents), 4861 (20.34%) medium producers (between 2 and 9 documents), and 18,800 (78.66%) small producers or occasional authors who have published a single document. The 10 most prolific authors in the field of diabetic foot are shown in Table 2, and their production over the last two decades analysed is shown in Figure 4.

There were 73 authors who published 20 or more papers on diabetic foot, within a total of 2481 papers. These documents were signed by 56 different institutions, including universities (33/73), health centres (31/73), and other kinds of institutions (9/73). The *Complutense University of Madrid* (Madrid, Spain) has the highest number of large producers with eight different authors who contributed 287 documents. The *Institutu Klinické is followed by Experimentální Medicíny* (Prague, Czech Republic), which contributed 118 documents produced by four different authors. And the centre *West China School of Medicine/West China Hospital of Sichuan University* (Chengdu, China) contributed a total of 89 documents signed by three authors. When looking at the production by country of these 73 authors, we observe that 15 of them were from the United States, and they contributed 583 documents, followed in productivity ranking by 10 authors from Spain, who contributed 354 documents, and 6 authors from United Kingdom, who contributed 280 documents. Table S3, in Supplementary Materials, includes a list of the institutions of these 73 prolific authors in production order.

#### 3.2.2. Author Collaboration Networks

**Figure 5:**
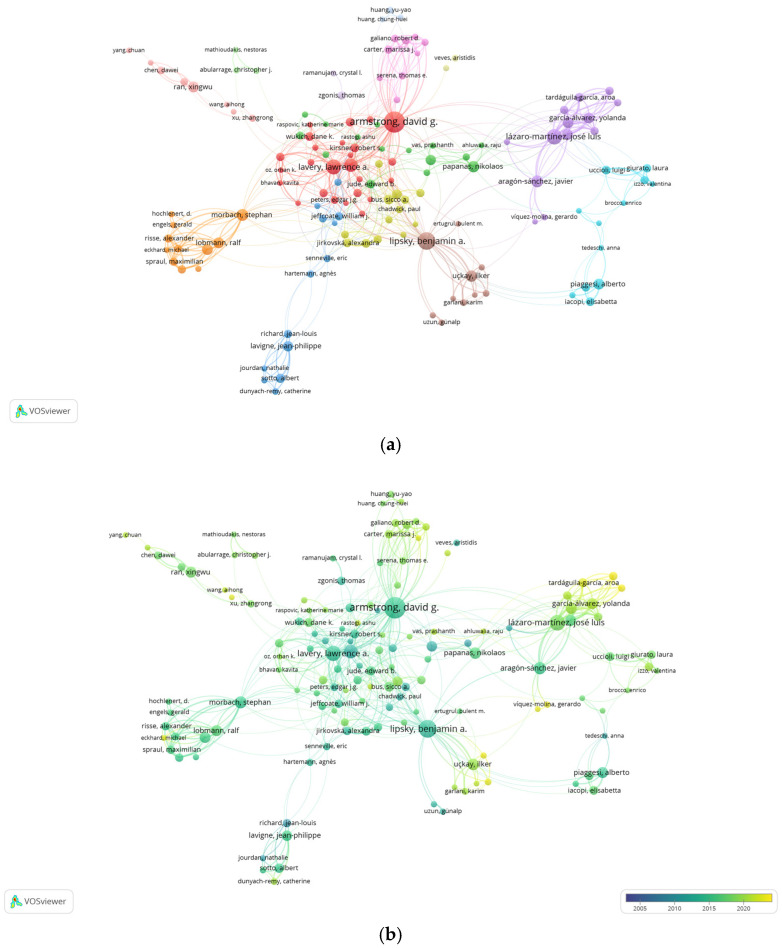
Collaboration networks between authors: (**a**) collaboration network of authors with 10 or more published documents and a maximum of 10 signatures per article on diabetic foot showing the 14 clusters generated and (**b**) collaboration network via overlay visualisation representing the production of these authors over time. The colours of the nodes, explained via a colour bar in the corner, show the current production of these authors, with yellow representing the most recent publications and blue representing the less recent ones, throughout the period of analysis. The node size represents the output of each author, and the thickness of the lines indicates the strength of association between authors.

The relationships established between authors with 10 or more publications with a maximum of 10 signatories per article are shown in Figure 5. In this network, 14 clusters of collaborations were generated between 139 authors. In descending order, the authors that present the largest nodes, that is, with the highest productivity, in these 14 clusters are as follows: 1. Armstrong D.G. (United States), 2. Lipsky B.A. (United Kingdom), 3. Lázaro-Martínez J.L. (Spain), 4. Morbach S. (Germany), 5. Papanas N. (Greece), 6. Ran X. (China), 7. Piaggessi A. (Australia), 8. Lavigne J.P. (France), 9. Bus S.A. (Netherlands), 10. Zgonis T. (United States), 11. Carter M.J. (United States), 12. Veves A. (United States), 13. Huang Y.-Y. (Taiwan), and 14. Abularrage C. (United States). The distribution by clusters of the 139 authors is shown in Supplementary Materials in Table S4.

### 3.3. Country Analysis

#### 3.3.1. Analysis of Countries Production and Citation

For the country analysis, data were exported through the Biblioshiny application, accounting for a total of 114 countries (35,269 occurrences). Table 3 shows the top 10 most productive and cited countries in diabetic foot research in the study period.

**Figure 6:**
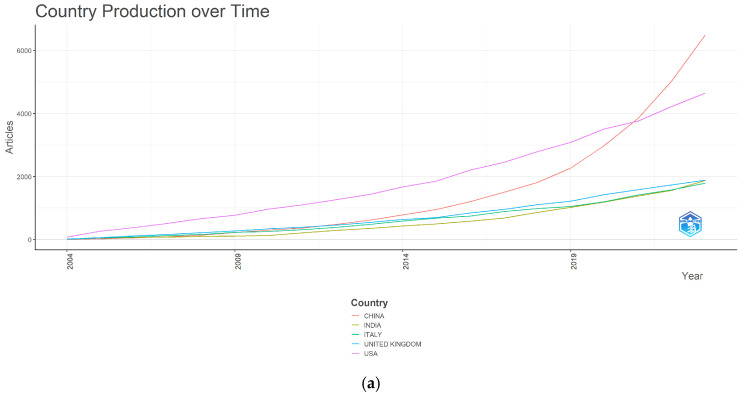

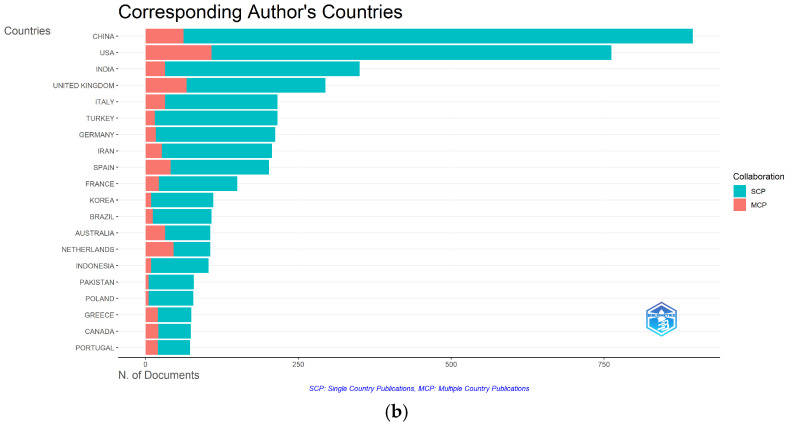
Publications by countries in the field of diabetic foot (2004–2023). (**a**) Country production over time; (**b**) collaboration between countries according to the affiliation of the corresponding author (SCPs: Single-Country Publications; MCPs: Multiple-Country Publications).

Through the graphs provided by Biblioshiny, we can see in Figure 6 the production over time in the five most productive countries (China; United States; United Kingdom; India; and Italy), where China’s scientific production has spiked in recent years (Figure 6a). The degree of collaboration between the top 20 countries according to the affiliation of the author chosen for correspondence is also showed, differentiating Single-Country Publications (SCPs) from Multiple-Country Publications (MCPs), clearly revealing that the countries with the greatest international collaboration are the United States and the United Kingdom, and the countries with the least collaboration are Pakistan and Poland (Figure 6b).

#### 3.3.2. Collaboration Networks Between Countries

**Figure 7:**
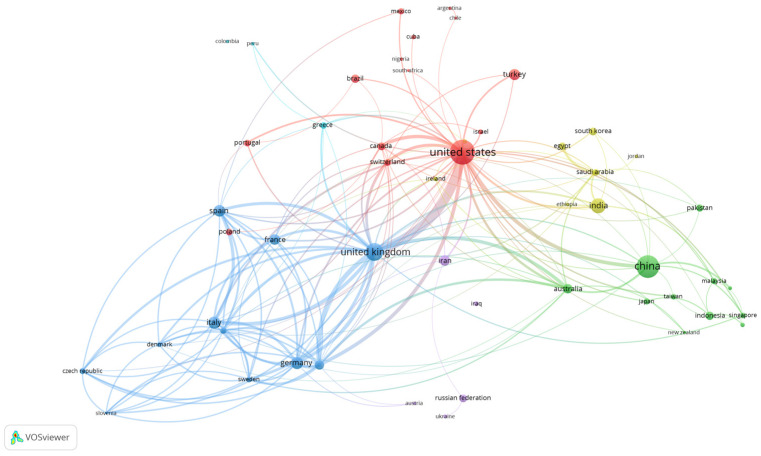
Network of international collaboration between countries. It shows the collaboration of 52 countries with at least 20 documents published on the world map. Each node represents a country/region, and each line represents a link between two countries/regions. The size of each node represents the number of documents, and the thickness of each line represents the strength of the link.

The map of country co-authorships generated through VOSviewer in Figure 7 shows the relationships between the 52 countries with at least 20 publications. These are grouped into six clusters, where it is clearly observed that the most powerful countries are the United States, United Kingdom, and China. Due to the thickness of the lines of union between nodes, the strong relations between the United States and the United Kingdom and between the member countries of the European Union are very well observed.

### 3.4. Journal Analysis

In the 7136 documents analysed on diabetic foot, a total of 1809 journals were counted, and we see that when ordering the journals by decreasing sequence of productivity and dividing them into equal parts, according to Bradford’s law, a small number of journals accumulate the highest production [54], the “core” of diabetic foot knowledge. The distribution of the different areas of Bradford is shown in Table 4, and a list of the 38 journals that make up the core of knowledge with their main bibliometric indicators are presented in the Supplementary Materials of this article in Table S5. All these journals belong to the medical and health sciences field and are scientific journals that are subject to peer review processes; they are mostly published in English and indexed in diverse bibliographic databases (citation indexes, multidisciplinary and specialised). Out of these 38 journals, 32 are indexed in the JCR database, 13 are published as open access, 5 are not open access, and the rest provide the reader with different proportions of free reading. The main editorial and access characteristics of these 38 journals, together with the JIF (Journal Impact Factor) with and without self-citations of JCR (2023) and their dissemination in databases, are also presented in Supplementary Materials in Table S6.

The top 10 most productive journals are shown in Table 5, and the top 10 most cited journals are shown in Table 6.

The journal with the highest number of publications on diabetic foot is the *International Journal of Lower Extremity Wounds* (278 publications), standing out from the rest, with almost four times the production of the one in position 10 in the ranking, which is *Diabetic Medicine* (72 publications). The journal which received the higher number of citations is *Diabetes Care* (8992 citations), in position 9 in the ranking of the most productive journals. Among the most cited, we find three journals (*Lancet*; *Clinical Infectious Diseases*; *Diabetologia*) in zone 1 according to Bradford’s distribution mentioned above. The journal *Lancet*, with only six publications, is ranked fourth in the ranking of the most cited journals. Most of the journals in these top 10 belong to the endocrinology and dermatology fields.

### 3.5. Most Cited and Referenced Documents

The analysed documents in our sample have received a total of 145,688 citations. Table 7 presents the top 10 most cited documents in the field of diabetic foot, and it can be observed that these documents were published from 2004 to 2017.

Regarding the references used by researchers in their publications, we have counted a total of 177,762. Table 8 presents the 10 most used bibliographic references in the set of documents analysed, which were published in the period running from 1981 to 2017.

### 3.6. Keyword Analysis

#### 3.6.1. Distribution of the Most Frequent Keywords

**Figure 8:**
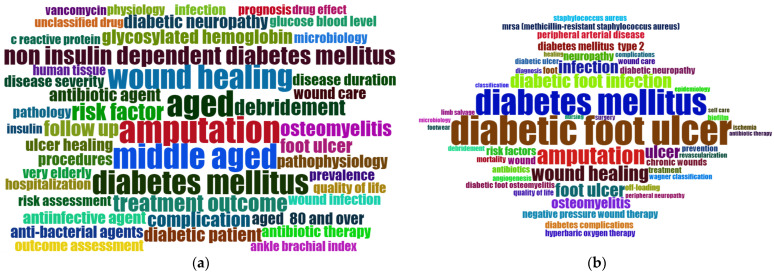
Most relevant keywords: (**a**) 50 index keywords (IDs); (**b**) 50 author keywords (DEs).

The keywords identified by the researchers were analysed through the *Index Keywords* (IDs) and *Author Keywords* (DEs) fields. Figure 8 shows the clouds of the 50 most relevant keywords in both categories, generated with the Biblioshiny application, after the removal of very general terms, unification of other equivalent terms through thesauri, and hiding the main term “diabetic foot” to facilitate a clearer visualisation of the terms of interest.

Among the most relevant keywords of both groups, apart from the terms related to the identification of diabetes mellitus and derived terms, the term “amputation”, as the main complication of the diabetic foot, and “wound healing” undoubtedly stand out. The terms related to infection are also striking, with osteomyelitis being one of the most worrying. There are numerous references to treatments, especially antibiotic treatment and major infectious agents. The term “quality of life” is represented in both groups.

Among the IDs, a more homogeneous distribution of the frequency of the terms is observed, and numerous terms related to the age of the diabetic population are evident, especially those referring to the elderly population. The 10 most frequent IDs were “amputation” (2380), “aged” (2225), “middle aged” (2145), “wound healing” (1914), “diabetes mellitus” (1863), “non-insulin dependent diabetes mellitus” (1144), “treatment outcome” (1002), “risk factor” (979), “debridement” (894), and “follow up” (821)”.

Among DEs, there is a greater prominence of diabetic foot ulcers, with the term “ulcer” being repeated both alone and as part of different keywords and terms related to infection and different treatments for wound healing. The 10 most frequent DEs were “diabetic foot ulcer” (1773), “diabetes mellitus” (1317), “amputation” (663), “wound healing” (447), “diabetic foot infection” (401), “foot ulcer” (393), “infection” (336), “ulcer” (333); “osteomyelitis” (251), and “neuropathy” (145).

#### 3.6.2. Co-Occurrence Analysis of Index Keywords

**Figure 9:**
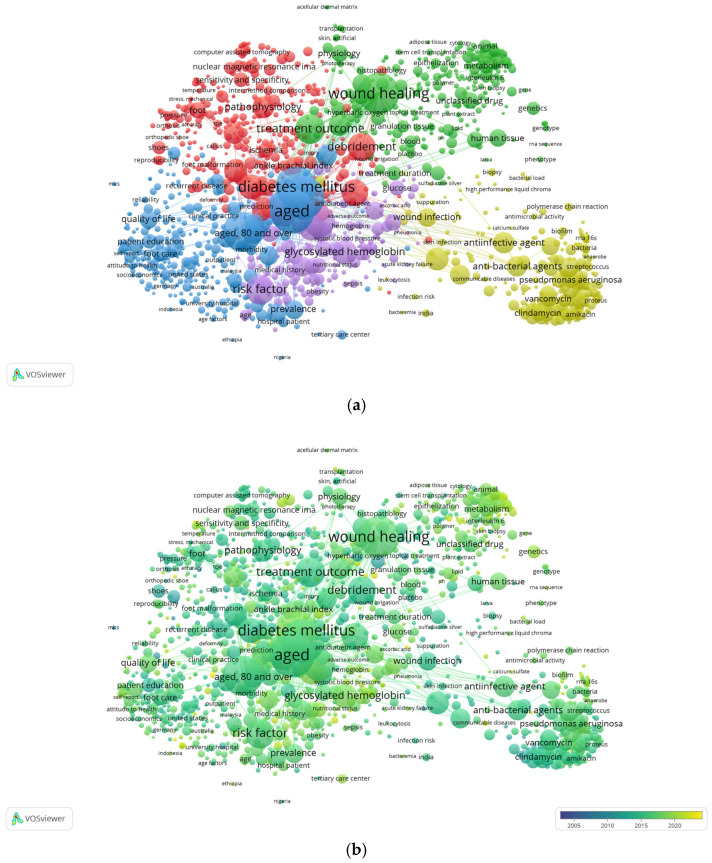
Co-occurrence networks of index keywords (IDs) of the 1413 IDs that appeared at least 15 times. (**a**) Co-occurrence network and (**b**) co-occurrence temporal overlap network maps. The colours of the nodes represent the ID groups. Each node represents a keyword and its co-occurrence size. Each line represents a link between two keywords and their thickness, that is, the strength of the link. In the co-occurrence time overlap map, the colour of each node represents the average publication year. The lighter colours show greater relevance of the terms, and the darker ones show greater age in the period analysed, 2004–2023.

Figure 9 shows the five clusters generated with the co-occurrences of index keywords (a) and the temporality in the appearance of the terms that we can observe with the variation in colours in the overlay view of the generated network (b).

By observing the network of co-occurrences generated between index keywords (IDs) that had a minimum frequency of repetition of 15 times (Figure 9a), we have identified the following five lines of research:

- *Cluster 1 (red): Diabetic foot diagnoses and complications.* This cluster of 336 IDs brings together key concepts on the general diagnostic and therapeutic management of diabetic foot, as well as concepts related to complications and vascular pathology. The 10 most representative terms (number of occurrences) are “debridement” (894), “osteomyelitis” (801), “foot ulcer” (684), “diabetic neuropathy” (619), “pathophysiology” (533), “procedures” (499), “infection” (391), “peripheral occlusive artery disease” (372), “ankle brachial index” (331), and “foot” (291).
- *Cluster 2 (green): Therapeutic management of diabetic foot ulcers.* This encompasses 321 IDs related to cell therapy and therapies that promote ulcer healing, including negative pressure therapy, and terms associated with experimental studies. The 10 most frequent terms in this cluster are “wound healing” (1914), “treatment outcome” (1002), “ulcer healing” (528), “pathology” (413), “human tissue” (353), “physiology” (337), “unclassified drug” (311), “drug effect” (300); “vacuum assisted closure” (288), and “metabolism” (270).
- *Cluster 3 (blue): Epidemiology and patient education and care.* In this, 312 IDs related to epidemiological data were counted, as well as numerous terms related to health services and patient education and care, quality of life, and where nursing is prevalent. The concept of “amputation” is also integrated into this group. The 10 most representative terms are “aged” (2225), “middle aged” (2145), “diabetes mellitus” (1863), “amputation” (1781), “diabetic patient” (672), “aged, 80 and over” (500), “wound care” (470), “outcome assessment” (436), “very elderly” (411), and “prevalence” (408)”.
- *Cluster 4 (yellow): Infections and anti-infective treatment.* This cluster of 261 IDs is slightly separated from the rest, constituting a very delimited subject in which we find numerous terms mainly related to infection, infectious agents, and antibiotic treatments. The 10 most frequent terms are “antibiotic agent” (599), “antiinfective agent” (513), “anti-bacterial agents” (483), “antibiotic therapy” (455), “staphylococcus aureus” (451), “wound infection” (398), “microbiology” (370), “vancomycin” (309), “pseudomonas aeruginosa” (298), and “ciprofloxacin” (296).
- *Cluster 5 (purple): Systemic complications and medical treatment.* In this cluster, we have counted 183 IDs associated with analytical parameters, as well as terms related to cardiovascular and renal pathology. The terms are related to risk factors and prognostic factors of diabetes mellitus and their possible relationship with a patient’s death. The most representative terms are “non-insulin dependent diabetes mellitus” (1114), “risk factor” (979), “follow up” (821), “complication” (731), “glycosylated hemoglobin” (691), “disease duration” (457), “disease severity” (446), “glucose blood level” (366), “prognosis” (335), and “insulin” (322).

With regard to the temporality of the IDs, through Figure 9b, in the overlay view of the network, we observe that the most recent terms, in lighter colours, respond to a multifaceted approach in which we can highlight, as an example, terms related to the pandemic (“pandemic”, “COVID-19”, “coronavirus disease 2019”), surgical management (“surgical debridement”, “amputation, surgical”), ratios and measures (“wound healing rate”, “ulcer healing rate”, “healing rate”, “Charlson comorbidity index”), genetics and cell biology (“exome”, “gene ontology”, “umbilical vein endothelial cell”, “long untranslated rna”, “differential gene expression”), infectious agents (“multidrug resistant bacterium”, “staphylococcus aureus infection”), and new technologies (“deep learning”, “machine learning”, “artificial intelligence”, “bioinformatics”), among others.

#### 3.6.3. Co-Occurrence Analysis of Author Keywords

**Figure 10:**
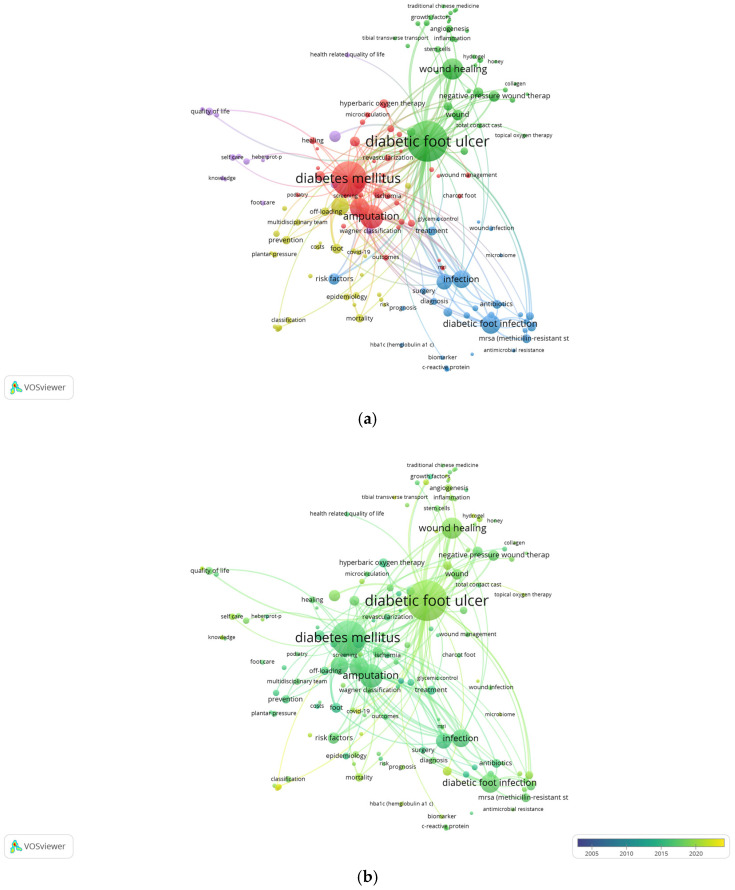
Co-occurrence networks of author keywords (DEs) of the 156 DEs that appeared at least 15 times. (**a**) Co-occurrence network and (**b**) co-occurrence temporal overlap network maps. The colours of the nodes represent the DE groups. Each node represents a keyword and its co-occurrence size. Each line represents a link between two keywords and their thickness, that is, the strength of the link. In the co-occurrence time overlap map, the colour of each node represents the average publication year. The lighter colours show greater relevance of the terms, and the darker ones show greater age in the period analysed, 2004–2023.

The author keywords (DEs) have also generated five clusters, in this case with 156 terms that have a minimum frequency of 15 repetitions. Figure 10 shows maps of the co-occurrences and a superimposed map that indicates the temporality of the DEs used in the publications analysed.

The lines of research generated are similar to the previous ones, although with some exceptions. After analysing the relationships established between the terms, we have identified the following:

- *Cluster 1 (red): Diabetic foot diagnoses and complications.* This cluster is similar to the ID one, including terms that cover the diagnosis and general treatment of diabetic foot as well as its complications. With 38 terms representing this cluster, the top 10 most frequent terms (number of occurrences) are “diabetes mellitus” (1317), “amputation” (605), “foot ulcer” (393), “neuropathy” (145), “peripheral arterial disease” (120), “diabetes complications” (105), “diabetic neuropathy” (104), ”hyperbaric oxygen therapy” (85), “revascularization” (57), and “limb salvage” (53).
- *Cluster 2 (green): Therapeutic management of diabetic foot ulcers.* This cluster contains 37 terms, the 10 most frequent being “diabetic foot ulcer” (1773), “wound healing” (447), “wound” (131), “negative pressure wound therapy” (123), “chronic wounds” (109), “wound care” (76), “diabetic ulcer” (74), “angiogenesis” (61), “debridement” (60), and “stem cells” (43).
- *Cluster 3 (blue): Infections and anti-infective treatment.* Made up of 34 DEs, this highlights terms related to infections, their treatments, and antibiotic resistance in the diabetic foot, as well as their risk factors. The 10 most representative terms are “diabetic foot infection” (401), “infection” (336), “osteomyelitis” (251), “risk factors” (143), “antibiotics” (91), “mrsa (methicillin-resistant staphylococcus aureus)” (89), “diabetic foot osteomyelitis” (83), “treatment” (83), “staphylococcus aureus” (74), and “biofilm” (66).
- *Cluster 4 (yellow): Epidemiology and prevention of ulcers.* This cluster contains 29 terms associated with epidemiology, prevention, diagnostic methods, and technologies based on artificial intelligence, and they reflect the importance of a multidisciplinary team for the treatment of diabetic foot. The most prominent are “ulcer” (333), “foot” (103), “prevention” (82), “off-loading” (76), “mortality” (75), “epidemiology” (50), “footwear” (49), “classification” (45), “multidisciplinary team” (43), and “prevalence” (43).
- *Cluster 5 (purple): Care and quality of life.* With 18 terms, the importance of quality of life, complications, self-care, and nursing care associated with this pathology are highlighted. The 10 most representative terms are “diabetes mellitus, type 2” (143), “quality of life” (72), “Wagner Classification” (63), “complications” (56), “self care” (46), “nursing” (44), “foot care” (29), “nursing care” (28), “depression” (26), and “health related quality of life” (26).

Regarding the most innovative DEs (Figure 10b) within our study period, which are emphasised in lighter colours, the relevant terms are “COVID-19”, “machine learning”, “deep learning”, “convolutional neural network”, “tibial transverse transport”, “self-management”, “hydrogel”, “validity”, “vitamin d”, “platelet rich plasma”, “diabetic foot osteomyelitis”, “exercise”, “microbiome”, and “reliability”, among others.

#### 3.6.4. Analysis of the Thematic Evolution of Author Keywords by Decade

To determine the thematic evolution of research on diabetic foot, the Biblioshiny application was used (with the same thesauri used in word clouds and keyword co-occurrence networks) to generate thematic maps that allow the extraction of groups of keywords whose density and centrality can be used to classify topics and represent them through quadrants in a two-dimensional diagram.

**Figure 11:**
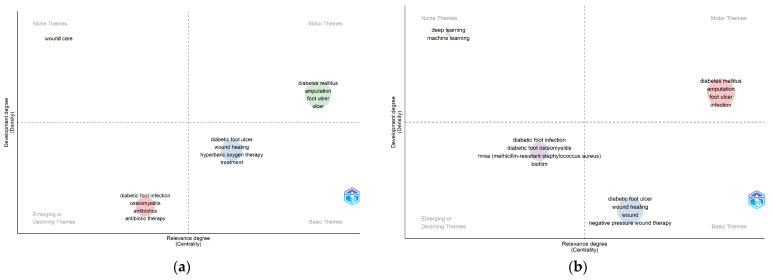
Maps of the thematic evolution by decades of research on diabetic foot according to the author keywords generated through the Walktrap grouping logarithm with 500 terms: (**a**) 2004–2013 period; (**b**) 2014–2023 period.

The quadrant of this diagram is formed by two coordinates indicating the centrality or degree of relevance (horizontal axis) and the density or degree of development (vertical axis). Thus, the groups with the highest values of centrality and density are the *motor or burning topics* (upper-right quadrant), which are those that represent advanced, well-developed and relevant knowledge, intensely researched in the field. The groups with the highest values of centrality and the lowest values of density are the *basic or transversal topics* (lower-right quadrant) fundamental to the field. Groups with lower values of centrality and density are defined as *emergent or peripheral topics* (lower-left quadrant), which are topics that are not fully developed or are marginally interesting for the research area. And the groups with the lowest values of centrality and the highest values of density are defined as *niche topics* (upper-left quadrant); they are topics that are developed but still marginal in the area of research [42,55]. To analyse the thematic evolution of research on diabetic foot, we have used only the author keywords because we consider them more representative of what the authors wanted to express in their publications, and Figure 11 shows the thematic evolution obtained.

## 4. Discussion

The results of this study allow us to update and compare the results of previous studies with similar characteristics to ours [18,25,33] that have also analysed global trends in research on diabetic foot.

### 4.1. General Publication Trends for the Diabetic Foot

We have analysed a total of 7136 documents indexed in the Scopus database that are published in 28 different languages. The majority (82.54%) are published in English, which is the most widespread language in the international scientific literature [37]. Publishing in English is beneficial as it allows researchers to reach a wider audience and thus have more options to obtain higher citation rates [30].

We found that, over the period analysed, scientific production on diabetic foot has grown exponentially, which has also been reflected in other studies [9,24] with which we compared [18,25,33]. Previous global bibliometric studies on diabetic foot have shown that scientific output has doubled its size worldwide from 2007 to 2017 and tripled from 2010 to 2020 [24]. The average number of documents published per year is 357 documents, a figure somewhat lower than that obtained in the study carried out by Zhao et al. [25] on global research on diabetic foot using the Web of Science database, where they obtained an average of 447 papers/year in the period 1998–2020. This difference could be due to the use of a different time period for their analysis and by the bibliographic database they used.

In relation to researchers in the field of diabetic foot, we have counted a total of 23,898 authors, who have signed their publications with an average of 5.27 ± 3.40 authors per document. These data are not provided in the studies with which we compared, but they are similar to the results obtained in other disciplines of health sciences, such as those obtained in the 70-year bibliometric analysis by Lutnick et al. [56] in 2021 on orthopaedic journals: the year 2019 presented an average of 5.7 authors per paper. Most of them are works published in collaboration (98.45%) where there is evidence of a very high level of international collaboration. Only 0.99% of the authors (237) are large producers, with 10 or more published papers, and 78.66% (18,800) are occasional authors who have published a single paper. It is estimated that the more consolidated and specialised an area is, the lower the rate of transience or occasional authors is [57,58]. The high percentage of occasional authors obtained in our study suggests that the diabetic foot field is still in a development and expansion phase, attracting contributions from a wide range of researchers and health professionals. We consider that this diversity of authors is enriching as they can bring new perspectives and innovative approaches to the study of the diabetic foot. However, the high percentage of occasional authors could also indicate a lack of specialisation sustained over time and a possible fragmentation of knowledge in the field as indicated by Nolan et al. [32] in their literature review of publication patterns of diabetic foot disease literature using the PubMed database. The presence of a large number of occasional authors could reflect the multidisciplinary nature of diabetic foot management, which involves specialists from various fields such as endocrinology, vascular surgery, podiatry, dermatology, and internal medicine, among others. However, this finding also underscores the need to encourage greater continuity in research and to establish specialised training programs to create a stronger base of experts dedicated exclusively to this field. Specialty consolidation could lead to more cohesion and, in the long term, potentially improve the quality and impact of diabetic foot studies.

The publication of evidence-based practical guidelines on the prevention and treatment of diabetic foot disease, such as the one led by the IWGDF (International Working Group on the Diabetic Foot) and carried out by multidisciplinary experts from around the world, contributes to better knowledge and management of diabetic patients by the global community of health professionals involved in their care [6]. These guides, which are updated annually and translated into different languages are reference documents that can help these health professionals to prevent and successfully treat diabetic foot disease [59] with an outcome of better ulcer prevention, amputation rate reduction, and helping to save lives, as well as reducing the economic costs of patient care.

In our study, the most influential authors who have contributed the most to the knowledge of the diabetic foot are Armstrong D.G. of the United States; Lipsky B.A. from the United Kingdom; and Lázaro-Martínez J.L. from Spain. The most cited and influential are, by far, Armstrong D.G. (13,481 citations) and Lipsky B.A. (9638 citations). The Spanish author Lázaro-Martínez J.L., appearing in third position in the production ranking, is seventh in the citation ranking with 1844 citations, so we observe that the most productive authors are not always the most cited. Lázaro-Martínez J.L. is an author who began his scientific production in the field of the diabetic foot in 2007, so this lower citation rate could be considered normal at the present time. Comparing our data with similar studies [25,33], we see a coincidence with the most productive and cited author, which is Armstrong D.G., but we find a difference with the authors in second and third position in the production and citation ranking, adding in their works Lavery L.A., Boulton A.J.M., and Bus S.A. From the co-authorship networks generated in our analysis we have been able to observe that, in general, in each cluster the most productive or influential author and his/her co-authors belong to the same geographical area.

Among the most productive countries/regions in the period analysed, China (18.40%), the United States (13.17%), and the United Kingdom (5.34%) stand out, data which coincide with what is reflected in bibliometric studies similar to ours that analyse world production [18,25,33]. These same three countries are the ones that received the most citations, although not in the same order. In the top 10 most cited countries in our study, the Netherlands stands out as the country with the highest average number of citations per document at 54.60, followed by the United States at 48.70. The United States is the most influential country, and the one that has received the most citations by far compared to the rest (28.58%); the United Kingdom received 8.97% and China 8.82% of the citations. It should also be noted that China has excelled in scientific production in recent years [37], an observation that was also pointed out by Zhao et al. [25] in their 2023 study. The United States and the United Kingdom have shown themselves to be the ones with the highest degree of international collaboration. China has presented a very high percentage of publications with exclusively national authors, a figure that coincides with the results obtained in a study on nursing care in the diabetic foot carried out by Zhao et al. [31] in 2024, where they state that China has limited collaboration with other countries despite its large production. The network of collaboration generated between the most productive countries shows us again that these three countries are the most powerful and that the United States and the United Kingdom show the strongest relations with the greatest centrality. The countries of the European Union, due to the size of their nodes and the thickness of the lines they generate in this network, also show great collaborative relationships between them. These countries have a very strong research infrastructure, relative to other countries or regions, which potentially allows them greater scientific output [30]. This distribution reflects a concentration of diabetic foot research in countries with a high prevalence of diabetes and advanced health systems, highlighting the global nature of this field of study and the importance of international collaboration in diabetic foot research.

In relation to the production and citation of the journals, in our sample we counted a total of 1809 journals, while the core of knowledge on diabetic foot was concentrated in 38 journals (2.10% of the total). The journals that generated the most publications were the *International Journal of Lower Extremity Wounds* (278 publications), with the *International Wound Journal* (210) and the *Journal of Wound Care* (158) standing out above the rest, coinciding with the data from Deng et al. [33] in 2022. In our analysis, the most productive journal was placed in the fifth position in the citation ranking, so we evidence, similarly with the authors, that the most prolific are not always the most cited. The journal that accumulated the highest number of citations was the magazine of the first quartile, *Diabetes Care*, located in the ninth position of the production ranking. And this leadership is also reflected in other bibliometric studies related to the diabetic foot [19,24,25,27,33]. In the study by Zhao et al. [25], *Diabetes Care* is also the most cited, but we did not obtain the same result for the top three most cited journals that they identified. In our study, the journals *Diabetes Medicine* and *Diabetology* are in the top seven and eight with the highest citations, respectively, instead of the second and third positions obtained by them.

Citation frequency is commonly used as an indicator of the quality and impact of published research. And this frequency of citation can be influenced by factors such as the perceived quality of the document, the quality of the journal, the relevance of the topic, the accessibility of the document, and the time cited [24]. The higher the degree of activity in an area, the lower the chance that an old publication will be cited, since the number of recent publications will be much higher than the number of old publications [60]. The time interval of the most cited documents in our study is established between 2004 and 2017, the most cited being the one published in 2017 by Armstrong, Boulton, and Bus, titled “Diabetic foot ulcers and their recurrence” in the *New England Journal of Medicine*, which received a total of 2245 citations. This outcome coincides with what was pointed out in the study by Deng et al. [33] The most cited articles are mostly of the review type and belong to journals located in quartile 1 of their thematic category. The most consulted reference among diabetic foot researchers in our study is the one published in the journal *JAMA* in 2005 entitled “Preventing foot ulcers in patients with diabetes” by Singh, Armstrong, and Lipsky. In the bibliometric study by Zhao et al. [25] conducted with the Web of Science database, that reference was, however, the third most cited reference.

### 4.2. Lines of Research of Greatest Interest in the Field of Diabetic Foot

In order to identify the most current lines of research and those that are under development, in our study we have analysed both author keywords and index keywords. On a general level, the most relevant terms include terms relating to amputation, wound healing, infection, and treatment, especially antibiotic treatment, as well as terms relating to infectious agents. Other frequent and relevant terms found were those related to the patient’s quality of life and to care through a multidisciplinary team. Within this multidisciplinary team, the nursing professional plays a very important role as numerous terms related to these professionals were detected, and this is because nursing plays a fundamental role in the care of diabetic foot ulcers. The nursing professional is responsible in many occasions for the daily care and management of wounds; they identify early symptoms and implement personalised care plans that prevent the appearance of many complications in diabetic patients [31].

In the co-occurrence networks generated with keywords that appeared at least 15 times, we have identified in our analysis five clusters in both the index keyword (ID) and author keyword (DE) categories. We detected three common lines that are related to diagnosis and complications, therapeutic management of ulcers and infections, and their treatment. In addition, the cluster of the IDs related to epidemiology and patient education and care correspond to the themes of the remaining two clusters of the DEs that we have identified as “epidemiology and prevention of ulcers” and “care and quality of life”. The cluster “systemic complications and medical treatment” was the only one exclusive to the IDs. The study by Deng et al. [33] used the same tool for network visualisation as the one we used (VOSviewer) and analysed the network of co-occurrences of the IDs with at least five occurrences. They also identified five other clusters, which in their case were grouped into studies of “rehabilitation”, “surgery”, “complications”, “molecular mechanisms”, and “clinical studies”. Their themes do not exactly coincide with ours, and this can be considered normal, since their network is configured with a number of different keyword occurrences and, in addition, they do not indicate that they used any thesauri to eliminate or join terms as we did in our analysis. In the study by Zhao et al. [25] using data from the Web of Science and the CiteSpace visualisation tool, they identified the following as the main themes in global research on diabetic foot: “diabetic wound healing”, “diabetic polyneuropathy”, “plantar pressure”, “diabetic foot infection”, “endovascular treatment”, and “hyperbaric oxygen therapy”. Zha et al. [18], with these same tools, identified “diabetic foot management”, “lower extremity amputation”, and “diabetic foot infection” as prominent topics.

Among the most innovative terms identified in both groups of keywords, in our study, we highlight terms referring to the COVID-19 pandemic; terms related to new technologies such as “artificial intelligence”, “deep learning”, “machine learning”, “convolutional neural network”, and “bioinformatics”; terms referring to genetics and cell biology such as “exome”, “gene ontology”, and “umbilical vein endothelial cell”; terms related to infections and infectious agents such as “diabetic foot osteomyelitis”, “multidrug resistant bacterium”, “staphylococcus aureus infection”, and “microbiome”, and terms related to new therapies such as “platelet-rich plasma”, among others. In the bibliometric study carried out by Deng et al. [33], mentioned above, using the VOSviewer tool, they noted that, within the five groups or lines of research they detected, the one that contained the largest number of most current keywords in the period 2004–2020 was the one related to the investigation of the molecular mechanism of diabetic foot ulcers. And in the study by Zha et al. [18], using the CiteSpace tool, the authors identified 15 keywords that represented the most current lines of research in the period 2007–2018; these were “resistant staphylococcus aureus”, “ulcer”, “expression”, “film”, “revascularization”, “peripheral neuropathy”, “management”, “foot ulceration”, “amputation”, “endothelial cell”, “diabetic foot ulcer”, “randomized controlled trial”, “multicenter”, “infection”, “plantar ulcer”, and “angipars”. Comparing these keywords with those obtained in our results, as the most novel terms in the period 2004–2023, we observe some synonyms and that infections and infectious agents continue to be a hot and current topic for research in the diabetic foot field [18].

The thematic maps generated try to represent how researchers perceive and communicate the topics of interest in the field of diabetic foot [55]. The thematic evolution that we have identified in the analysis of author keywords evidence that the hottest topics throughout the 20 years of analysis are those related to diabetes mellitus, amputations, ulcers, and diabetic foot infections. As basic topics, the ones which stand out are aspects related to the healing of ulcers with treatments such as hyperbaric oxygen therapy and, more recently, negative pressure wound therapy. Among the emerging topics, we observed that diabetic foot infection, osteomyelitis, and antibiotic treatments stood out, with a major role played by antibiotic resistance. As niche topics, wound care in the first decade and terms related to artificial intelligence such as deep learning and machine learning have gained greater interest by researchers in recent years. The emergence of new technologies associated with artificial intelligence promises to revolutionise diabetic foot care by providing greater precision in diagnosis and in the planning of personalised treatment strategies, taking advantage of advances in medical imaging, biomarker detection, and clinical biomechanics [61].

Diabetic foot remains a major challenge despite medical advances [31]. Since complications of diabetic foot disease can be difficult to manage, preventing new or recurring complications should be a key priority [5]. Diabetic foot management includes early diagnosis, well-coordinated multidisciplinary care [4], prompt treatment of infection and peripheral artery disease, as well as optimal wound and discharge care [5]. Multidisciplinary teams are essential in the comprehensive approach to the diabetic foot, acting mainly in four areas: glycemic control, local wound management, vascular disease, and infection [62]. Multidisciplinary care achieves higher wound healing rates and lower levels of major lower limb amputation [32,62].

This comprehensive approach is also reflected in the diversity of topics and disciplines represented in the publications analysed in our bibliometric study. The results show a growing trend towards collaborative research among different specialties, which underlines the importance of a holistic approach in the treatment of diabetic foot, where there is a team including different professionals who are experts in different specialties, with the sole objective of delving into the patient’s pathology. The prominence of institutions and authors from diverse countries focused on diabetic foot disease in our analysis suggests a coordinated global effort to address this complex complication of diabetes. In addition, the evolution of publications over time indicates a growing emphasis on preventive strategies and the development of innovative technologies for early diagnosis and effective treatment. This trend toward a more proactive and technologically advanced approach to diabetic foot management promises to improve clinical outcomes and reduce the socioeconomic burden associated with this chronic patient condition.

### 4.3. Strength and Limitations

We consider as strengths of our study the data update in relation to bibliometric studies already published, comparable to ours, which have analysed trends worldwide [18,25,33]. We have used a bibliographic database and data analysis tools different from those used by others in their studies. We have based our analysis on Scopus, which has greater documentary and idiomatic coverage [48], while Zha et al. [18], Zhao et al. [25], and Deng et al. [33] used the Web of Science database. For the analysis and visualisation of the data, they used the CiteSpace tool [18,25] and VOSviewer [33], while we have combined Biblioshiny and VOSviewer for a more complete analysis. In addition, we incorporated new results and added an independent analysis of index keywords and author keywords to avoid missing useful information in the thematic analysis of this research front.

We also note that our study has some limitations that should be considered when interpreting the results. We only used the Scopus database to export data, and although its coverage is large and it is a reference for bibliometric studies, it may be not exhaustive and may omit relevant publications indexed in other sources or in languages not covered by this database. In addition, our search was based on a single descriptor, “diabetic foot”, and was executed exclusively in the title field for a better adjustment of the theme; this fact could have excluded relevant research that used alternative terminology or those that actually address the topic did not reflect the term in the title. On the other hand, despite efforts to standardise data both automatically and manually, inconsistencies in the way authors, countries, or institutions are cited in the original publications may have affected the accuracy of our counts and analyses.

The lack of data normalisation and ambiguity in the names of authors and institutions is a common problem that greatly hinders the recovery of their publications and their recognition, limiting their visibility in the scientific community [60]. It is essential that authors become aware of the problems that this lack of normalisation can entail, both with their own names when signing their papers and with the names of the institutions to which they belong [63]. Moreover, it would be desirable that all scientific journals include mechanisms to standardise the identification of researchers who publish in their journals, as currently not all of them include these policies or control mechanisms; this would greatly improve the reliability and quality of the data used for many bibliometric studies.

The limitations noted underline the need to interpret the results with caution, suggesting that future studies could benefit from a broader approach in terms of searching, the inclusion of multiple databases, and the analysis of the quality and methodology used in the research to offer an even more comprehensive view of the field of diabetic foot research. Despite these limitations, we consider that the results obtained in our study provide a solid basis for understanding the current state of research in the field of diabetic foot and may help to guide future directions of study, collaboration, and resource allocation.

## 5. Conclusions

This bibliometric study describes the characteristics of publications indexed in Scopus over the last 20 years (2004–2023) on diabetic foot, providing a comprehensive view of the global research landscape in this field. The analysis reveals significant trends in scientific production, identifying the most relevant topics, the most prolific authors, and the most influential countries and publications that have contributed substantially to the advancement of diabetic foot knowledge. The results obtained not only reflect the growing global interest in this complication of diabetes but also highlight the evolution of international collaboration and the impact of research in different regions. The importance of diabetic foot has led to the production of a large number of scientific publications, and while we acknowledge that it is a widely studied subject, and while we acknowledge that it is a widely studied subject, we consider that it is still in the process of development and expansion because we observe that a wide range of research and health professionals contribute to the field. This multidisciplinary care enriches diabetic foot care and is essential for the comprehensive approach to this type of patient.

## Figures and Tables

**Table 1:** A summary of the characteristics of the documents.

	Description	Result
Main information about data	Timespan	2004–2023
	Journals	1809
	Documents	7136
	Annual growth rate (%)	10.44
	Document average age	7.55
	Average citations per document	20.4
	Total citations received	145,688
	Total references	177,762
Document types	Article (%)	5956 (83.46)
	Review (%)	1180 (16.54)
	Open access (%)	2938 (41.17)
	Open-access article (%)	2518 (85.70)
	Open-access review (%)	420 (14.30)
	Publication languages	28
Authors	Authors (occurrences)	23,898 (37,604)
	Authors of single-author documents	440
Author collaboration	Single-author document	582
	Co-authors per document	5.27
	International co-authorships (%)	13.66
	Countries (occurrences)	114 (35,269)
Document contents	Documents with index keywords (%)	6105 (85.55)
	Index keywords (occurrences)	177,031
	Mean ± standard deviation per document	24.81 ± 17.42
	Documents with author keywords (%)	5366 (75.20)
	Author keywords (occurrences)	27,255
	Mean ± standard deviation per document	3.82 ± 2.11

**Table 2:** Top 10 most productive authors in the field of diabetic foot (2004–2023).

Rank	Author (ID Scopus) * Affiliation More Recent in Scopus	TPs (PF)	TCs	Local h-Index	FAP (%p)
1	Armstrong, David G. (7404407396) Keck School of Medicine of USC, Los Angeles (United States)	160 (31.69)	13,481	54	33 (20.63)
2	Lipsky, Benjamin A. (7006768971) Green Templeton College, Oxford (United Kingdom)	108 (25.61)	9638	49	18 (16.67)
3	Lázaro-Martínez, José Luis (18434633300) Universidad Complutense de Madrid, Madrid (Spain)	86 (14.99)	1844	21	17 (19.77)
4	Lavery, Lawrence A. (7006066609) UT Southwestern Medical School, Dallas (United States)	76 (14.17)	6311	37	25 (32.89)
5	Boulton, Andrew J.M. (7202295225) University of Miami, Miami (United States)	61 (21.55)	8555	38	14 (24.95)
6	Edmonds, Michael (16439677500) King’s College Hospital, London (United Kingdom)	57 (17.02)	4250	29	14 (24.56)
7	Piaggesi, Alberto (7004496777) Monas University, Melbourne (Australia)	51 (7.57)	3220	20	10 (19.61)
8	Morbach, Stephan (6603754743) Marien Krankenhaus, Soest (Germany)	49 (10.50)	1004	13	24 (48.98)
9	Aragón-Sánchez, Javier (6507768519) La Paloma Hospital, Las Palmas de Gran Canaria (Spain)	47 (14.46)	1552	20	26 (55.32)
10	Ran, Xingwu (35269932400)West China School of Medicine/West China Hospital of Sichuan University (China)	45 (8.70)	546	11	2 (4.44)

* Affiliation (last available in Scopus October 2024)—Abbreviations: ID: Scopus Author Identifier; TPs: total publications; PF: publications fractionalised; TCs: total citations; local h-index: h-index of the sample obtained through Bibliometrix; FAP: first author position; %p: percentage that represents the total production of the author (TPs).

**Table 3:** Top 10 most productive countries and top 10 most cited countries in the field of diabetic foot (2004–2023).

Rank	Country Production	Freq. (%)	Rank	Country Most Cited	TCs (%)	ADCs
1	China	6488 (18.40)	1	United States	37,144 (28.58)	48.70
2	United States	4645 (13.17)	2	United Kingdom	11,653 (8.97)	39.60
3	United Kingdom	1885 (5.34)	3	China	11,463 (8.82)	12.80
4	India	1877 (5.32)	4	Netherlands	6847 (5.27)	54.60
5	Italy	1788 (5.07)	5	India	6611 (5.09)	18.90
6	Turkey	1266 (3.59)	6	Italy	4266 (3.28)	19.80
7	Spain	1257 (3.56)	7	France	3998 (3.08)	26.70
8	Iran	1230 (3.49)	8	Germany	3847 (2.96)	18.10
9	Germany	1115 (3.16)	9	Iran	3751 (2.98)	18.10
10	France	1067 (3.03)	10	Turkey	3033 (2.33)	14.00

Abbreviations: Freq. (%): frequency and percentage out of a total of 35,269 country occurrences; TCs (%): total citations and percentage out of a total of 129,946 citations received; ADCs: average document citations.

**Table 4:** Distribution of journals and documents by Bradford’s areas.

Zone	Journals (%)	Documents (%)
Core	38 (2.10)	2363 (33.11)
Zone 1	267 (14.76)	2377 (33.31)
Zone 2	1504 (83.14)	2396 (33.58)

Percentage out of 1809 journals and 7136 documents.

**Table 5:** Top 10 most productive journals in the field of diabetic foot (2004–2023).

Rank	Journal	TPs	TCs	Local h-Index	Category	Quartile (2023)	SJR (2023)	h-Index (2023)
1	*International Journal of Lower Extremity Wounds*	278	4405	32	Surgery	Q2	0.43	49
2	*International Wound Journal*	210	5614	42	Dermatology	Q1	0.73	83
3	*Journal of Wound Care*	158	2244	27	Fundamentals and Skills	Q2	0.4	77
4	*Wounds*	96	1077	20	Medical and Surgical Nursing	Q2	0.3	49
5	*Diabetes Research and Clinical Practice*	96	2802	33	Endocrinology	Q1	1.34	140
6	*Diabetes/Metabolism Research and Reviews*	94	5869	42	Endocrinology	Q1	1.99	135
7	*Journal of the American Podiatric Medical Association*	85	1426	22	Podiatry	Q3	0.2	65
8	*Wound Repair and Regeneration*	84	2924	32	Dermatology	Q1	0.8	133
9	*Diabetes Care*	79	8992	55	Advanced and Specialized Nursing	Q1	5.69	418
10	*Diabetic Medicine*	72	3634	32	Endocrinology	Q1	1.3	165

Abbreviations: TPs: total publications; TCs: total citations; local h-index: sample h-index value; category: category ranked in the best quartile according to the Scimago Journal and Country Rank (SJR) in 2023; data for quartile, SJR, and h-index are from SJR in 2023.

**Table 6:** Top 10 most cited journals in the field of diabetic foot (2004–2023).

Rank	Journal	PR	TCs	TPs	Local h-Index	Category	Quartile (2023)	SJR (2023)	h-Index (2023)
1	*Diabetes Care*	9	8992	79	55	Advanced and Specialized Nursing	Q1	5.69	418
2	*Diabetes/Metabolism Research and Reviews*	6	5869	94	42	Endocrinology	Q1	1.99	135
3	*International Wound Journal*	2	5614	210	42	Dermatology	Q1	0.73	83
4	*Lancet*	220	5372	6	6	Medicine (miscellaneous)	Q1	12.11	895
5	*International Journal of Lower Extremity Wounds*	1	4405	278	32	Surgery	Q2	0.43	49
6	*Clinical Infectious Diseases*	79	3733	14	14	Infectious Diseases	Q1	3.31	387
7	*Diabetic Medicine*	10	3634	72	32	Endocrinology	Q1	1.3	165
8	*Diabetologia*	48	3461	21	18	Endocrinology	Q1	3.36	261
9	*Wound Repair and Regeneration*	8	2924	84	32	Dermatology	Q1	0.8	133
10	*Diabetes Research and Clinical Practice*	5	2802	96	33	Endocrinology	Q1	1.34	140

Abbreviations: PR: productivity ranking; TCs: total citations; TPs: total publications; local h-index: sample h-index value; category: category ranked in the best quartile according to the Scimago Journal and Country Rank (SJR) in 2023; data for quartile, SJR, and h-index are from SJR in 2023.

**Table 7:** Top 10 most cited articles in the field of diabetic foot (2004–2023).

Rank	Most Cited Articles	TCs	ST	Quartile (2023)	SJR (2023)
1	Armstrong et al. Diabetic foot ulcers and their recurrence. *New England Journal of Medicine* (2017)—doi: 10.1056/NEJMra1615439	2245	Review	Q1	20.54
2	Falaga V. Wound healing and its impairment in the diabetic foot. *Lancet* (2005)—doi: 10.1016/S0140-6736(05)67700-8	1874	Review	Q1	12.11
3	Boulton et al. The global burden of diabetic foot disease. *Lancet* (2005)—doi: 10.1016/S0140-6736(05)67698-2	1854	Review	Q1	12.11
4	Lipsky et al. 2012 infectious diseases society of America clinical practice guideline for the diagnosis and treatment of diabetic foot infections. *Clinical Infectious Diseases* (2012)—doi: 10.1093/cid/cis346	1348	Review	Q1	3.31
5	Zhang et al. Global epidemiology of diabetic foot ulceration: a systematic review and meta-analysis. *Annals of Medicine* (2017)—doi: 10.1080/07853890.2016.1231932	986	Review	Q1	1.31
6	Lipsky et al. Diagnosis and treatment of diabetic foot infections. *Clinical Infectious Diseases* (2004)—doi: 10.1086/424846	890	Review	Q1	3.31
7	Armstrong et al. Negative pressure wound therapy after partial diabetic foot amputation: A multicentre, randomised controlled trial. *Lancet* (2005)— doi: 10.1016/S0140-6736(05)67695-7	776	Article	Q1	12.11
8	Prompers et al. Prediction of outcome in individuals with diabetic foot ulcers: Focus on the differences between individuals with and without peripheral arterial disease. The Eurodiale study. *Diabetologia* (2008)—doi: 10.1007/s00125-008-0940-0	760	Article	Q1	3.36
9	Prompers et al. High prevalence of ischaemia, infection and serious comorbidity in patients with diabetic foot disease in Europe. Baseline results from the Eurodiale study. *Diabetologia* (2007)—doi: 10.1007/s00125-006-0491-1	755	Article	Q1	3.36
10	Fryberg et al. Diabetic foot disorders: a clinical practice guideline (2006 revision). *Journal of Foot and Ankle Surgery* (2006)—doi: 10.1016/S1067-2516(07)60001-5	614	Article	Q1	0.7

Abbreviations: TCs: total citations; ST: study type; SJR: Scimago Journal and Country Rank.

**Table 8:** Top 10 most cited references.

Rank	Most Cited References	Citations
1	Singh N., Armstrong D.G., Lipsky B.A., Preventing foot ulcers in patients with diabetes, *JAMA*, 293, 2, Pp. 217–228, (2005)—doi: 10.1001/jama.293.2.217	553
2	Armstrong D.G., Boulton A.J.M., Bus S.A., Diabetic foot ulcers and their recurrence, *N Engl J Med*, 376, 24, Pp. 2367–2375, (2017)—doi: 10.1056/NEJMra1615439	322
3	Boulton A.J., Vileikyte L., Ragnarson-Tennvall G., Apelqvist J., The Global Burden of Diabetic Foot Disease, *Lancet*, 366, Pp. 1719–1724, (2005)—doi: 10.1016/S0140-6736(05)67698-2	319
4	Jeffcoate W.J., Harding K.G., Diabetic foot ulcers, *Lancet*, 361, 9368, Pp. 1545–1551, (2003)—doi: 10.1016/S0140-6736(03)13169-8	170
5	Lavery L.A., Armstrong D.G., Wunderlich R.P., Mohler M.J., Wendel C.S., Lipsky B.A., Risk factors for foot infections in individuals with diabetes, *Diabetes Care*, 29, 6, Pp. 1288–1293, (2006)—doi: 10.2337/dc05-2425	165
6	Falaga V., Wound healing and its impairment in the diabetic foot, *Lancet*, 366, 9498, Pp. 1736–1743, (2005)—doi: 10.1016/S0140-6736(05)67700-8	151
7	Wagner F.W., The dysvascular foot: A system for diagnosis and treatment, *Foot Ankle*, 2, Pp. 64–122, (1981)—doi: 10.1177/107110078100200202	102
8	Armstrong D.G., Lavery L.A., Harkless L.B., Validation of a diabetic wound classification system. The contribution of depth, infection, and ischemia to risk of amputation, *Diabetes Care*, 21, Pp. 855–859, (1998)—doi: 10.2337/diacare.21.5.855	95
9	Moulik P.K., Mtonga R., Gill G.V., Amputation and mortality in new-onset diabetic foot ulcers stratified by etiology, *Diabetes Care*, 26, Pp. 491–494, (2003)—doi: 10.2337/diacare.26.2.491	83
10	Lipsky B.A., Osteomyelitis of the foot in diabetic patients, *Clin Infect Dis*, 25, Pp. 1318–1326, (1997)— doi: 10.1086/516148	72

## Data Availability

The original contributions presented in this study are included in this article/Supplementary Materials. Further inquiries can be directed to the corresponding author.

## Supplementary Materials

The following supporting information can be downloaded at https://www.mdpi.com/article/10.3390/ijerph22040463/s1, Table S1: BIBLIO checklist for the bibliometric review of the medical literature, indicating the pages in which the items of our article are framed; Table S2: Distribution of the languages of publication of documents on diabetic foot in the period 2004–2023; Table S3: Affiliations of authors with 20 or more papers per order of production; Table S4: Cluster distribution of the 139 authors that make up the co-authorship network; Table S5: Journals conforming the core of knowledge on diabetic foot according to Bradford’s areas; and Table S6: Main editorial characteristics, access characteristics, JCR impact indicators (2023), and dissemination in databases of the journals that form the core of knowledge on diabetic foot according to Bradford’s areas.
