# Supplementary material for: Global Trends in Diabetic Foot Research (2004–2023): A Bibliometric Study Based on the Scopus Database
Source: Int J Environ Res Public Health. 2025 Mar 21;22(4):463. doi: 10.3390/ijerph22040463 (PMC12026514; doi:10.3390/ijerph22040463)
Supplement: Supplementary file 1 [file ijerph-22-00463-s001.zip › NewSupplementary Materials_ijerph-3461218_2025.03.20.pdf]

Contents of Supplementary Materials:

Table S1: BIBLIO checklist for the bibliometric review of the medical literature, indicating the pages in which the items of our article are framed.

Table S2: Distribution of the languages of publication of documents on diabetic foot in the period 2004-2023.

Table S3: Affiliations of authors with 20 or more papers per order of production.

Table S4: Cluster distribution of the 139 authors that make up the co-authorship network.

Table S5: Journals conforming the core of knowledge on diabetic foot according to Bradford's areas.

Table S6: Main editorial characteristics, access characteristics, JCR impact indicators (2023), and dissemination in databases of the journals that form the core of knowledge on diabetic foot according to Bradford's areas.

Table S1: BIBLIO checklist for the bibliometric review of the medical literature, indicating the pages in which the items of our article are framed.

Section/Topic	Item No.	Checklist item	Reported on page No.
**Title**			
Identification	1	Identify the report as a bibliometric review in the title.	P.1 (Title)
Issues/topics	2	Indicate the key issues/topics under investigation and coverage of time period.	P.1 (Title and Keywords)
**Abstract**			
Structured summary	3	Structured summary including (as applicable): background, methods, results (key findings) and conclusions.	P.1 (Abstract)
**Introduction/ Background**			
Justification/ Rationale/ Explanation	4	Present review of existing knowledge and epidemiological information.	Pp.1-2 (Introduction)
Objectives	5	Statement of the objective (s) or question (s).	P.2 (Introduction)
**Methods**			
Search engines (data sources)	6	Describe all information sources (such as electronic databases, contact with study authors, trial registers or other grey literature sources).	P.3 (Methods)
Search strategy	7	Keywords and systematization criteria (date of search, language, type of document) for the search.	P.3 (Methods)
Time period	8	The period that the review covers and the justification.	P.3 (Methods)
Eligibility criteria	9	Describe all inclusion and exclusion criteria; languages; study design, type of publication and time period.	P.3 (Methods)
Data refinement (data selection procedure)	10	Remove the irrelevant articles; inspection to eliminate duplicate and unrelated articles (after evaluation of the title, abstract and content).	Pp.3-4 (Methods))
Quality assessment (optional)	11	Assessment of papers by three authors and the use of assessing checklists.	Pp.3-4 (Methods))
Data synthesis	12	Describe the methods used for summarizing, handling, synthesis, tabulations or schematic displays. Describe how the data were analysed.	Pp.4-5 (Methods))
**Results**			
Descriptive findings (statistics)	13	- Provide details of the search and selection process in a flow diagram.<br>- Number of citations retrieved (number of publications, year of publication, type of documents, country of publication, articles with	P.4 (Methods)<br><br>Pp.5-21 (Results))

		the highest impact, most impactful authors, most impactful articles, authors with the highest production, top journals, top institutions, …)	
Schematic map and trend	14	Summarize and/or present the schematic maps and trends using an appropriate software to present citations, journals, authors, top journals, time trends, emerging literature, and any relevant indicators (as applicable) [1-5].	Pp.5-21 (Results)
Tabulation and summarizing the findings	15	General recommendation: Studies under consideration could be summarized and organized by different subtitles and different scenarios. Regardless, results need to be presented in separate tables covering each subtitle. The followings are some options that could help to summarize the findings.<br><br>*Option 1:*<br>- Start the presentation with a historical view [when and who first published on the topic].<br>- Report on review papers. The result should be listed in a separate table. Also, specify the review type (scoping review, narrative review, systematic review, and meta-analysis).<br>- Summarize the findings according to the study designs and main study types.<br>*Option 2:*<br>- Start the presentation with a historical view [when and who first published on the topic].<br>- Report on review papers. The result should be listed in a separate table. Also, indicate the review type (scoping review, narrative review, systematic review, and meta-analysis) should be specified.<br>- Summarize the findings according to outcome measures or populations. For example, see [6].<br>*Option 3:*<br>- Start the presentation with a historical view [when and who first published on the topic].<br>- Report on review papers. The result should be listed in a separate table. Also, specify the review type (scoping review, narrative review, systematic review, and meta-analysis).<br>- Summarize the findings according to concept [7].<br>*Option 4.*<br>- Start the presentation with a historical view [when and who first published on the topic].<br>- Report on review papers. The result should be listed in a separate table, and also specify the review type (scoping review, narrative review, systematic review, and meta-analysis).	Pp.5-21 (Results)

		- Summarize the findings according to different subtitles relevant to the main topic [8].	
Synthesis of findings	16	Synthesize the findings as much as possible, find the gap, and propose a model, hypothesis, etc. (if applicable).	
**Discussion**			
Summary of evidence	17	Summarize the main findings. The findings should be presented in more "general" or "accessible" terms.	Pp.22-25 (Discussion)
Interpretation	18	Include interpretation consistent with results. Explanations for observed outcomes, similarities, and differences reported would be essential.	Pp.22-25 (Discussion)
Strengths and limitations	19	Discuss the strengths and limitations.	Pp.25-26 (Discussion)
Conclusion(s)	20	Provide a general interpretation of the results with respect to the review questions and objectives, as well as potential implications.	P.26 (Conclusions)

1. McDougal L, Dehingia N, Cheung WW, Dixit A, Raj A. COVID-19 burden, author affiliation and women's well-being: A bibliometric analysis of COVID-19 related publications including focus on low-and middle-income countries. eClinicalMedicine 2022; 52: 101606.
2. Henstock L, Wong R, Tsuchiya A, Spencer A. Behavioral theories that have influenced the way health state preferences are elicited and interpreted: A bibliometric mapping analysis of the ttime trade-off method with VOSviewer visualization. Front Health Serv 2022; 2: 848087.
3. Bodea F, Bungau SG, Negru AP, Radu A, Tarce AG, Tit DM, et al. Exploring new therapeutic avenues for ophthalmic disorders: Glaucoma-related molecular docking evaluation and bibliometric analysis for improved management of ocular diseases. Bioengineering 2023; 10(8): 983.
4. Sang XZ, Wang CQ, Chen W, Rong H, Hou LJ. An exhaustive analysis of post-traumatic brain injury dementia using bibliometric methodologies. Front Neurol 2023; 14: 1165059.
5. Ramli MI, Hamzaid NA, Engkasan JP, Usman J. Respiratory muscle training: a bibliometric analysis of 60 years' multidisciplinary journey. Biomed Eng Online 2023; 22(1): 50.
6. Akosman I, Kumar N, Mortenson R, Lans A, De La Garza Ramos R, Eleswarapu A,et al. Racial differences in perioperative complications, readmissions, and mortalities after elective spine surgery in the United States: A systematic review using AI-assisted bibliometric analysis. Glob Spine J 2023: 21925682231186759.
7. Tavousi M, Mohammadi S, Sadighi J, Zarei F, Kermani RM, Rostami R, Montazeri A. Measuring health literacy: A systematic review and bibliometric analysis of instruments from 1993 to 2021. Plos One 2022; 17(7): e0271524.
8. Montazeri A. Health-related quality of life in breast cancer patients: A bibliographic review of the literature from 1974 to 2007. J Exp Clin Cancer Res 2008; 27: 32.

Rights and permissions: The original source of the checklist is: Montazeri A, Mohammadi S, M.Hesari P, Ghaemi M, Riazi H, Sheikhi-Mobarakeh Z. Preliminary guideline for reporting bibliometric reviews of the biomedical literature (BIBLIO): a minimum requirements. *Systematic Reviews* 2023; 12: 239. doi.org/10.1186/s13643-023-02410-2 The article is licensed under a Creative Commons Attribution 4.0 International License (http://creativecommons.org/licenses/by/4.0/). A changes was made to the original checklist to add in full references to the cited sources.

Table S2: Distribution of the languages of publication of documents on diabetic foot in the period 2004-2023.

Rank	Languages	Num. Documents	Percentage
**1**	English	5890	82.54
**2**	Chinese	369	5.17
**3**	German	183	2.56
**4**	Russian	179	2.51
**5**	Spanish	144	2.02
**6**	French	94	1.32
**7**	Czech	41	0.57
**8**	Ukrainian	41	0.57
**9**	Japanese	41	0.57
**10**	Turkish	26	0.36
**11**	Portuguese	24	0.34
**12**	Persian	22	0.31
**13**	Italian	15	0.21
**14**	Polish	12	0.17
**15**	Korean	9	0.13
**16**	Slovak	9	0.13
**17**	Croatian	7	0.10
**18**	Romanian	5	0.07
**19**	Hungarian	4	0.06
**20**	Dutch	4	0.06
**21**	Greek	4	0.06
**22**	Hebrew	4	0.06
**23**	Arabic	2	0.03
**24**	Bosnian	2	0.03
**25**	Bulgarian	2	0.03
**26**	Finnish	1	0.01
**27**	Danish	1	0.01
**28**	Azerbaijani	1	0.01
	**Total:**	**7136**	**100.00**

Table S3: Affiliations of authors with 20 or more papers per order of production.

Rank	Author (ID Scopus)	TPs	PF	Affiliation more recent in Scopus	City	Country	Type of affiliation
1	Armstrong, David G. (7404407396)	160	31.69	Keck School of Medicine of USC	Los Angeles	United States	Educational institution
2	Lipsky, Benjamin A. (7006768971)	108	25.61	Green Templeton College	Oxford	United Kingdom	Educational institution
3	Lázaro-Martínez, José Luis (18434633300)	86	14.99	Universidad Complutense de Madrid	Madrid	Spain	Educational institution
4	Lavery, Lawrence A. (7006066609)	76	14.17	UT Southwestern Medical School	Dallas	United States	Educational institution
5	Boulton, Andrew J.M. 7202295225)	61	21.55	University of Miami	Coral Gables	United States	Educational institution
6	Edmonds, Michael (16439677500)	57	17.02	King's College Hospital	London	United Kingdom	Health Center
7	Piaggesi, Alberto (7004496777)	51	7.57	Monash University	Melbourne	Australia	Educational institution
8	Morbach, Stephan (6603754743)	49	10.5	Marien Krankenhaus, Soest	Soest	Germany	Health Center
9	Aragón-Sánchez, Javier (6507768519)	47	14.46	La Paloma Hospital	Las Palmas de Gran Canaria	Spain	Health Center
10	Ran, Xingwu (35269932400)	45	8.7	West China School of Medicine/West China Hospital of Sichuan University	Chengdu	China	Educational institution /Health Center
11	Papanas, Nikolaos (12763313600)	44	11.68	Democritus University of Thrace	Komotini	Greece	Educational institution
12	Jude, Edward B. (7003521236)	43	6.95	Tameside and Glossop Integrated Care NHS Foundation Trust	Ashton-under-Lyne	United Kingdom	Health Center
13	Lobmann, Ralf (6701744779)	43	12.54	Klinikum Stuttgart	Stuttgart	Germany	Health Center
14	Uccioli, Luigi (56015453000)	43	5.99	CTO Andrea Alesini	Rome	Italy	Health Center
15	Uçkay, Ilker (22935926900)	42	8.21	Uniklinik Balgrist	Zurich	Switzerland	Health Center
16	García-Álvarez, Yolanda (36236825300)	39	6.53	Universidad Complutense de Madrid	Madrid	Spain	Educational institution

Rank	Author (ID Scopus)	TPs	PF	Affiliation more recent in Scopus	City	Country	Type of affiliation
17	Jirkovská, Alexandra (57197802703)	38	6.13	Institutu Klinické a Experimentální Medicíny	Prague	Czech Republic	Health Center
18	Rümenapf, Gerhard (7004032315)	38	8.07	Deaconess Foundation Hospital	Speyer	Germany	Health Center
19	Apelqvist, Jan (7004430070)	37	7.22	Skånes Universitetssjukhus	Lund	Sweden	Health Center
20	García-Morales, Esther (18434169300)	37	6.28	Universidad Complutense de Madrid	Madrid	Spain	Educational institution
21	Lavigne, Jean-Philippe (24531297400)	37	6.12	Université de Montpellier	Montpellier	France	Educational institution
22	Álvaro-Afonso, Francisco Javier (55628422700)	36	6.23	Universidad Complutense de Madrid	Madrid	Spain	Educational institution
23	Bus, Sicco A. (7004095333)	34	9.83	Universiteit van Amsterdam	Amsterdam	Netherlands	Educational institution
24	Spraul, Maximilian (7004926811)	34	6.35	Diabetes Schwerpunktpraxis	Rheine	Germany	Health Center
25	van Netten, Jaap J. (23983869500)	33	6.84	Universiteit van Amsterdam	Amsterdam	Netherlands	Educational institution
26	Sotto, Albert (7006249763)	32	5.29	Centre Hospitalier Universitaire de Nîmes	Nimes	France	Health Center
27	Meloni, Marco (57215300329)	31	4.03	Università degli Studi di Roma "Tor Vergata"	Rome	Italy	Educational institution
28	Reike, Heinrich (6602994983)	31	4.79	Mariannen-Hospital Werl	Werl	Germany	Health Center
29	Schaper, Nicolaas C. (7004156278)	31	5.59	Universiteit Maastricht	Maastricht	Netherlands	Educational institution
30	Wukich, Dane K. (6602215301)	31	7.66	UT Southwestern Medical Center	Dallas	United States	Health Center
31	Fejfarová, Vladimíra (6602334784)	30	4.35	Institutu Klinické a Experimentální Medicíny	Prague	Czech Republic	Health Center
32	Frykberg, Robert G. (7003666198)	30	8.61	Fountain Hills	Fountain Hills	United States	Health Center
33	Kirsner, Robert S. (7101773124)	29	6.79	University of Miami Leonard M. Miller School of Medicine	Miami	United States	Educational institution
34	Iacopi, Elisabetta (50161569500)	28	4.42	Università di Pisa	Pisa	Italy	Educational institution

Rank	Author (ID Scopus)	TPs	PF	Affiliation more recent in Scopus	City	Country	Type of affiliation
35	Giurato, Laura (35933127700)	27	4.24	Università degli Studi di Roma "Tor Vergata"	Rome	Italy	Educational institution
36	van Baal, Jeff G. (7004075327)	27	4.29	Ziekenhuisgroep Twente	Almelo	Netherlands	Health Center
37	Bém, Robert (12139074100)	26	5.04	Institutu Klinické a Experimentální Medicíny	Prague	Czech Republic	Health Center
38	Larijani, Bagher (57205307740)	26	4.74	Endocrinology and Metabolism Research Institute (TUMS)	Tehran	Iran	Other
39	Lazzarini, Peter A. (41561537500)	26	4.71	Queensland University of Technology	Brisbane	Australia	Educational institution
40	Peters, Edgar J.G. (57217629913)	26	5.78	Amsterdam UMC - University of Amsterdam	Amsterdam	Netherlands	Educational institution
41	Veves, Aristidis (35595507100)	25	4.81	Harvard Medical School	Boston	United States	Educational institution
42	Zgonis, Thomas (6701546705)	25	8.83	The University of Texas Health Science Center at San Antonio	San Antonio	United States	Educational institution
43	Bowling, Frank L. (14062925300)	24	5.91	Manchester Royal Infirmary	Manchester	United Kingdom	Health Center
44	Dubský, Michal (56422302100)	24	2.97	Institutu Klinické a Experimentální Medicíny	Prague	Czech Republic	Health Center
45	Game, Frances (6701759105)	24	7.52	University Hospitals of Derby and Burton NHS Foundation Trust	Derby	United Kingdom	Health Center
46	Jeffcoate, William J. (57203073839)	24	5.16	Nottingham University Hospitals NHS Trust	Nottingham	United Kingdom	Health Center
47	La Fontaine, Javier (24168754900)	24	3.87	The University of Texas Rio Grande Valley	Brownsville	United States	Educational institution
48	Richard, J.-L. (55608085700)	24	6.03	Centre Hospitalier Universitaire de Nîmes	Nimes	France	Health Center
49	Risse, Alexander (55887966800)	24	4.69	Diabeteszentrum am Sophie-Charlotte-Platz	Berlin	Germany	Health Center
50	Senneville, Eric (7003355556)	24	7.78	Gustave Dron Hospital	Tourcoing	France	Health Center
51	Tardáguila-García, Aroa (57193884183)	24	4.08	Universidad Complutense de Madrid	Madrid	Spain	Educational institution
52	Malone, Matthew (55928381000)	23	3.79	Liverpool Hospital	Liverpool	Australia	Health Center

Rank	Author (ID Scopus)	TPs	PF	Affiliation more recent in Scopus	City	Country	Type of affiliation
53	Mohajeri-Tehrani, Mohammad Reza (15728245200)	23	3.31	Endocrinology and Metabolism Research Institute (TUMS)	Tehran	Iran	Other
54	Carter, Marissa J. (16834696100)	22	3.78	Strategic Solutions	Bozeman	United States	Other
55	Chen, Dawei (57148912500)	22	3.07	West China School of Medicine/West China Hospital of Sichuan University	Chengdu	China	Educational institution /Health Center
56	Goretti, Chiara (54883016100)	22	3.74	Università di Pisa	Pisa	Italy	Educational institution
57	López-Moral, Mateo (57210151169)	22	3.67	Universidad Complutense de Madrid	Madrid	Spain	Educational institution
58	Sanz-Corbalán, Irene (55556236600)	22	3.7	Universidad Complutense de Madrid	Madrid	Spain	Educational institution
59	Viswanathan, Vijay (55767770100)	22	5.8	M.V. Hospital for Diabetes	Chennai	India	Health Center
60	Wang, Chun (55860799369)	22	2.98	West China School of Medicine/West China Hospital of Sichuan University	Chengdu	China	Educational institution /Health Center
61	Xu, Zhangrong (55648766000)	22	7.11	PLA Strategic Support Force Characteristic Medical Center	Beijing	China	Health Center
62	Han, Seung-Kyu (26040694200)	21	4.8	Korea University Guro Hospital	Seoul	South Korea	Educational institution /Health Center
63	Hartemann, A. (36966232500)	21	6.07	Hôpital Universitaire Pitié Salpêtrière	Paris	France	Health Center
64	Molines-Barroso, Raúl J. (55773421900)	21	3.5	Universidad Complutense de Madrid	Madrid	Spain	Educational institution
65	Van Acker, Kristien (7006331968)	21	2.88	Centre de Santé des Fagnes Clinique Chimay	Chimay	Belgium	Health Center
66	Ahmad, Jamal (7102588381)	20	6.9	Aligarh Muslim University	Aligarh	India	Educational institution
67	Galiano, Robert D. (6603936643)	20	2.65	Northwestern University Feinberg School of Medicine	Chicago	United States	Educational institution
68	Mauricio, Didac (7004517251)	20	1.71	Universitat de Vic - Universitat Central de Catalunya (UVic-UCC)	Vic	Spain	Educational institution
69	Mills, Joseph L. (7402862404)	20	5.51	Baylor College of Medicine	Houston	United States	Educational institution
70	Müller, Eckhard (8124559900)	20	3.77	KfH-Nierenzentrum	Bernkastel-Kues	Germany	Health Center

Rank	Author (ID Scopus)	TPs	PF	Affiliation more recent in Scopus	City	Country	Type of affiliation
71	Serena, Thomas E. (8878304500)	20	3.21	SerenaGroup Research Foundation	Cambridge	United States	Other
72	Wu, Stephanie C. (9632686300)	20	5.4	Rosalind Franklin University of Medicine and Science	North Chicago	United States	Educational institution
73	Zelen, Charles M. (6506228440)	20	2.96	Professional Education and Research Institute	Roanoke	United States	Other

Abbreviations: TPs: total publications; PF: publications fractionalised.

Table S4: Cluster distribution of the 139 authors that make up the co-authorship network.

N	Cluster (N authors)	Author	Total link strength	Documents in cluster	Citations
1	1 (25 authors)	Armstrong, David G.	198	138	10,266
2		Lavery, Lawrence A.	159	70	5444
3		Boulton, Andrew J.M.	81	55	8266
4		Frykberg, Robert G.	22	26	818
5		Kirsner, Robert S.	28	26	1918
6		Wukich, Dane K.	36	26	937
7		Bowling, Frank L.	28	23	732
8		La Fontaine, Javier	60	22	678
9		Malone, Matthew	26	21	521
10		Peters, Edgar J.G.	49	20	1731
11		Wu, Stephanie C.	29	20	795
12		Lantis, John C.	10	15	541
13		Kim, Paul J.	35	14	553
14		Rajbhandari, Satyan	9	13	407
15		Attinger, Christopher E.	28	12	559
16		Vileikyte, Loretta	19	12	2865
17		Bhavan, Kavita	37	11	327
18		Crews, Ryan T.	17	11	344
19		Margolis, David J.	14	11	768
20		Oz, Orhan K.	31	11	238
21		Raspovic, Katherine Marie	18	11	386
22		Reeves, Neil D.	15	11	512
23		Steinberg, John S.	19	10	225
24		Van Asten, Suzanne A.V.	32	10	403
25		Vickery, Karen	10	10	403
26	2 (15 authors)	Papanas, Nikolaos	43	42	870
27		Edmonds, Michael	27	35	1473
28		Jude, Edward B.	44	26	745
29		Viswanathan, Vijay	7	20	591
30		Maltezos, Efstratios	20	17	528
31		Tentolouris, Nikolaos	11	15	468
32		Abbas, Zulfiqarali G.	10	14	542
33		Dhamodharan, Umapathy	11	13	312
34		Vas, Prashanth	14	13	304
35		Chockalingam, Nachiappan	7	11	185
36		Driver, Vickie R.	7	11	975
37		Formosa, Cynthia	4	11	144
38		Ahluwalia, Raju	19	10	206
39		Kesavan, Rajesh	12	10	325
40		Rastogi, Ashu	5	10	235
41	3 (15 authors)	Lavigne, Jean-Philippe	76	34	1076
42		Sotto, Albert	72	29	939
43		Richard, Jean-Louis	44	22	739
44		Jeffcoate, William J.	34	21	1759
45		Game, Frances	24	19	841
46		Senneville, Eric	15	18	1138
47		Hartemann, Agnès	6	17	571
48		Mills, Joseph L.	29	16	958

N	Cluster (N authors)	Author	Total link strength	Documents in cluster	Citations
49		Dunyach-Remy, Catherine	35	15	306
50		Schuldiner, Sophie	33	13	382
51		Boyko, Edward J.	18	12	1593
52		Jourdan, Nathalie	34	12	525
53		Monteiro-Soares, Matilde	18	10	898
54	4 (13 authors)	Bus, Sicco A.	40	32	4544
55		Van Netten, Jaap J.	48	30	1490
56		Fejfarová, Vladimíra	63	25	416
57		Jirkovská, Alexandra	60	23	395
58		Lazzarini, Peter A.	12	22	616
59		Apelqvist, Jan	21	21	3673
60		Bém, Robert	58	21	350
61		Dubský, Michal	57	19	363
62		Van Baal, Jeff G.	22	19	478
63		Schaper, Nicolaas C.	28	18	2057
64		Chadwick, Paul	11	16	370
65		Mrozikiewicz-Rakowska, Beata	2	12	105
66		Hinchliffe, Robert J.	13	10	945
67	5 (13 authors)	Lázaro-Martínez, José Luis	269	76	1524
68		Aragón-Sánchez, Javier	95	46	1538
69		García-Álvarez, Yolanda	169	39	384
70		García-Morales, Esther	144	36	922
71		Álvaro-Afonso, Francisco Javier	157	36	441
72		Tardáguila-García, Aroa	106	24	176
73		López-Moral, Mateo	96	22	73
74		Sanz-Corbalán, Irene	98	22	221
75		Molines-Barroso, Raúl J.	100	21	335
76		García-Madrid, Marta	78	18	68
77		Beneit-Montesinos, Juan Vicente	37	12	544
78		Víquez-Molina, Gerardo	23	12	71
79		López-Valverde, María Eugenia	21	10	70
80	6 (12 authors)	Piaggesi, Alberto	69	36	648
81		Uccioli, Luigi	62	26	726
82		Iacopi, Elisabetta	58	25	335
83		Giurato, Laura	56	22	562
84		Goretti, Chiara	55	22	250
85		Meloni, Marco	54	22	343
86		Coppelli, Alberto	38	13	201
87		Izzo, Valentina	37	11	223
88		Tedeschi, Anna	20	11	404
89		Anichini, Roberto	14	10	261
90		Brocco, Enrico	18	10	180
91		Seghieri, Giuseppe	9	10	245
92	7 (11 authors)	Morbach, Stephan	158	46	660
93		Lobmann, Ralf	71	37	860
94		Rümenapf, Gerhard	127	36	225
95		Risse, Alexander	115	23	120
96		Spraul, Maximilian	111	23	112
97		Reike, Heinrich	112	21	114
98		Müller, Eckhard	111	20	111
99		Engels, Gerald	18	13	83
100		Hochlenert, D.	16	12	66

N	Cluster (N authors)	Author	Total link strength	Documents in cluster	Citations
101		Lawall, Holger	6	12	144
102		Eckhard, Michael	65	11	26
103	8 (10 authors)	Lipsky, Benjamin A.	160	98	6715
104		Uçkay, Ilker	87	40	995
105		Gariani, Karim	37	15	380
106		Waibel, Felix W. A.	41	14	85
107		Uzun, Günalp	14	13	381
108		Mutluoglu, Mesut	15	12	317
109		Schöni, Madlaina	35	12	62
110		Ertugrul, Bulent M.	7	11	199
111		Kressmann, Benjamin	31	11	254
112		Berli, Martin C.	29	10	94
113	9 (9 authors)	Carter, Marissa J.	95	21	412
114		Didomenico, Lawrence A.	70	13	338
115		Galiano, Robert D.	92	19	386
116		Glat, Paul M.	60	11	99
117		Orgill, Dennis P.	90	17	322
118		Reyzelman, Alexander M.	27	15	602
119		Serena, Thomas E.	50	17	668
120		Snyder, Robert J.	20	13	462
121		Zelen, Charles M.	94	19	653
122	10 (8 authors)	Ran, Xingwu	51	39	243
123		Chen, Dawei	38	19	157
124		Wang, Chun	40	19	155
125		Xu, Zhangrong	13	17	188
126		Wang, Aihong	7	11	12
127		Yang, Chuan	1	11	90
128		Chen, Lihong	23	10	117
129		Chen, Mingwei	1	10	38
130	11 (3 authors)	Abularrage, Christopher J.	21	13	642
131		Hicks, Caitlin W.	21	12	731
132		Mathioudakis, Nestoras	20	11	1018
133	12 (3 authors)	Huang, Yu-Yao	23	14	331
134		Huang, Chung-Huei	20	10	274
135		Lin, Cheng-Wei	20	10	147
136	13 (2 authors)	Veves, Aristidis	1	15	868
137		Schmidt, Brian M.	3	13	109
138	14 (2 authors)	Zgonis, Thomas	16	23	350
139		Ramanujam, Crystal L.	12	12	105

Note: Authors with 10 or more published documents and a maximum of 10 signatures per article in the period 2004-2023 sorted by highest to lowest scientific production on diabetic foot. Data exported from the co-authoring network generated with VOSviewer.

Table S5: Journals conforming the core of knowledge on diabetic foot according to Bradford's areas.

Rank	Journal (ISSN)	TPs	% (7136)	H-Index (2023)	SJR (2023)	Quartile (2023)	Category
1	International Journal of Lower Extremity Wounds (15347346)	278	15.37	49	0.43	Q2	Surgery
2	International Wound Journal (17424801, 1742481X)	210	11.61	83	0.73	Q1	Dermatology
3	Journal of Wound Care (09690700, 20522916)	158	8.73	77	0.4	Q2	Fundamentals and Skills
4	Wounds (10447946, 19432704)	96	5.31	49	0.3	Q2	Medical and Surgical Nursing
5	Diabetes Research and Clinical Practice (01688227, 18728227)	96	5.31	140	1.34	Q1	Endocrinology
6	Diabetes/Metabolism Research and Reviews (15207552, 15207560)	94	5.20	135	1.99	Q1	Endocrinology
7	Journal of the American Podiatric Medical Association (19308264, 87507315)	85	4.70	65	0.2	Q3	Podiatry
8	Wound Repair and Regeneration (10671927, 1524475X)	84	4.64	133	0.8	Q1	Dermatology
9	Diabetes Care (01495992, 19355548)	79	4.37	418	5.69	Q1	Advanced and Specialized Nursing
10	Diabetic Medicine (07423071, 14645491)	72	3.98	165	1.3	Q1	Endocrinology
11	Advances in Skin and Wound Care (15277941, 15388654)	62	3.43	71	0.4	Q2	Advanced and Specialized Nursing
12	Chinese Journal of Diabetes Mellitus (16745809)	61	3.37	8	0.13	Q4	Endocrinology, Diabetes and Metabolism

Rank	Journal (ISSN)	TPs	% (7136)	H-Index (2023)	SJR (2023)	Quartile (2023)	Category
13	Journal of Clinical Medicine (20770383)	60	3.32	113	0.88	Q1	Medicine (miscellaneous)
14	Diabetes and Metabolic Syndrome: Clinical Research and Reviews (18714021, 18780334)	59	3.26	83	1.31	Q1	Endocrinology, Diabetes and Metabolism
15	Journal of Diabetes and its Complications (10568727, 1873460X)	58	3.21	98	1.02	Q1	Internal Medicine
16	PLoS ONE (19326203)	55	3.04	435	0.84	Q1	Multidisciplinary
17	Journal of Foot and Ankle Surgery (10672516, 15422224)	54	2.99	77	0.7	Q1	Surgery
18	Journal of Diabetes Research (23146745, 23146753)	51	2.82	83	0.99	Q2	Endocrinology
19	Journal of Foot and Ankle Research (17571146)	47	2.60	54	0.7	Q2	Orthopedics and Sports Medicine
20	Frontiers in Endocrinology (16642392)	44	2.43	120	1.24	Q1	Endocrinology, Diabetes and Metabolism
21	Diabetic Foot and Ankle (2000625X)	41	2.27	26	0	-	Podiatry / Internal Medicine
22	Foot 09582592, 15322963	40	2.21	44	0.36	Q2	Podiatry
23	Medicine (United States) (00257974, 15365964)	36	1.99	174	0.44	Q3	Medicine (miscellaneous)
24	Diabetes, Metabolic Syndrome and Obesity (11787007)	35	1.93	64	0.84	Q2	Internal Medicine
25	Diabetologe (18609716, 18609724)	33	1.82	0	0	-	Endocrinology, Diabetes and Metabolism
26	Klinichna khirurhiia / Ministerstvo okhorony zdorov'ia Ukraïny, Naukove tovarystvo khirurhiv Ukraïny (00232130)	32	1.77	6	0	-	Medicine (miscellaneous) / Surgery

Rank	Journal (ISSN)	TPs	% (7136)	H-Index (2023)	SJR (2023)	Quartile (2023)	Category
27	International Journal of Diabetes in Developing Countries (09733930)	32	1.77	33	0.25	Q3	Internal Medicine
28	Cochrane Database of Systematic Reviews (14651858)	32	1.77	327	1.41	Q1	Medicine (miscellaneous)
29	Clinics in Podiatric Medicine and Surgery (08918422, 15582302)	31	1.71	40	0.27	Q3	Orthopedics and Sports Medicine
30	Khirurgiia (Mosk) (00231207, 23095628)	30	1.66	13	0.22	Q3	Surgery
31	Journal of Vascular Surgery (07415214, 10976809)	29	1.60	218	1.94	Q1	Cardiology and Cardiovascular Medicine
32	Current Diabetes Reviews (15733998, 18756417)	29	1.60	68	0.77	Q2	Endocrinology
33	Journal of Diabetes Science and Technology (19322968)	28	1.55	93	1.05	Q1	Bioengineering
34	Zhongguo xiu fu chong jian wai ke za zhi = Zhongguo xiufu chongjian waike zazhi = Chinese journal of reparative and reconstructive surgery (10021892)	27	1.49	16	0.17	Q4	Medicine (miscellaneous)
35	Foot and Ankle International (10711007)	27	1.49	128	1.5	Q1	Orthopedics and Sports Medicine
36	Journal of Tissue Viability (0965206X, 18764746)	26	1.44	41	0.61	Q2	Dermatology
37	Journal of Diabetes and Metabolic Disorders (22516581)	26	1.44	46	0.6	Q2	Internal Medicine
38	Chinese Journal of Tissue Engineering Research (16738225)	26	1.44	12	0.12	Q4	Biomedical Engineering

Abbreviations: TPs: total publications; category: category ranked in the best quartile according to the Scimago and Journal and Country Rank (SJR) in 2023; data for h-index, SJR and quartile are from SJR in 2023.

Table S6: Main editorial characteristics, access characteristics, JCR impact indicators (2023), and dissemination in databases of the journals that form the core of knowledge on diabetic foot according to Bradford's areas.

Rank	Journal (Publisher; Region; Language)	Frequency (issues/year)	Journal OA	Total citable (%OA)	JIF (2023)	JIF without self citations (2023)	*Diffusion C/M/E databases (Num. databases)
1	International Journal of Lower Extremity Wounds (SAGE Publications Inc.; United States; English)	4	Not	341 (2.64%)	1.5	1.3	1, 2 / 6 / 10-12 (6)
2	International Wound Journal (Wiley; England; English)	6	Yes	899 (81.87%)	2.6	2.3	1, 2 / 5 / 10-14 (8)
3	Journal of Wound Care (MA Healthcare LTD; England; English)	12	Not	500 (0%)	1.5	1.3	1, 2 / - / 10-14 (7)
4	Wounds (HMP Communications; United States; English)	12	Not	211 (0%)	1.4	1.2	1, 2 / - / 11, 12 (4)
5	Diabetes Research and Clinical Practice (Elsevier Ireland LTD; Netherlands; English)	12	Not	1136 (23.50%)	6.1	5.9	1, 2 / 7 / 10-12, 15, 18 (8)
6	Diabetes/Metabolism Research and Reviews (Wiley; England; English)	8	Not	272 (39.71%)	4.6	4.3	1, 2 / - / 10-15 (8)
7	Journal of the American Podiatric Medical Association (American Podiatric Medical Association; United States; English)	6	Not	349 (0%)	0.5	0.5	1, 2 / - / 10-13 (4)
8	Wound Repair and Regeneration (Wiley; United States; English)	6	Not	210 (24.76%)	3.8	3.8	1, 2 / 7 / 10-13, 15 (8)
9	Diabetes Care (American Diabetes Association; Unites States; English)	12	Not	996 (1.2%)	14.8	14.2	1, 2 / 6-8 / 10-14, 16, 19 (12)
10	Diabetic Medicine (Wiley; England; English)	12	Not	578 (43.08%)	3.2	3.0	1, 2 / 7 / 10-14 (8)
11	Advances in Skin and Wound Care (Lippincott Williams & Wilkins; United States; English)	12	Not	319 (8.78%)	1.7	1.6	1-3 / - / 10-12, 14 (7)
12	Chinese Journal of Diabetes Mellitus (Chinese Medical Journals Publishing House Co.Ltd; China; Chinese)	12	Not	-	-	-	1 / - / - (1)
13	Journal of Clinical Medicine (MDPI; Switzerland; English)	24	Yes	20,573 (99.73%)	3.0	2.8	1, 2 / 7 / 11, 14 (5)
14	Diabetes and Metabolic Syndrome: Clinical Research and Reviews (Elsevier; India; English)	6	Not	651 (4.61%)	4.3	4.3	1, 4 / - / 11, 12, 17 (5)

Rank	Journal<br>(Publisher; Region; Language)	Frequency<br>(issues/year)	Journal OA	Total citable<br>(%OA)	JIF<br>(2023)	JIF without self citations<br>(2023)	*Diffusion<br>C/M/E databases<br>(Num. databases)
15	Journal of Diabetes and its Complications<br>(Elsevier SCI LTD; United States; English)	6	Not	475<br>(15.79%)	2.9	2.8	1, 2 / 7 / 10-12, 14-16<br>(9)
16	PLoS ONE<br>(Public Library Science; United States; English)	U	Yes	46,172<br>(99.34%)	2.9	2.8	1, 2/ 5, 7, 8 / 11-18, 20-26<br>(20)
17	Journal of Foot and Ankle Surgery<br>(Elsevier Science INC; United States; English)	6	Not	663<br>(5.43%)	1.3	1.2	1, 2 / - / 10-12<br>(5)
18	Journal of Diabetes Research<br>(Hindawi LTD; United States; English)	12	Yes	405<br>(99.75%)	3.6	3.6	1, 2 / 5, 7 / 11-14<br>(8)
19	Journal of Foot and Ankle Research<br>(BMC; England; English)	1	Yes	244<br>(100%)	2.5	2.2	1, 2 / 5, 7 / 11, 12, 14<br>(7)
20	Frontiers in Endocrinology<br>(Frontiers Media SA; Switzerland; English)	1	Yes	8233<br>(99.7%)	3.9	3.6	1, 2 / 5 / 11, 12, 15<br>(6)
21	Diabetic Foot and Ankle<br>(Taylor & Francis LTD; England; English)	1	Yes	8<br>(87.50%)	-	-	1, 4 / - / 14<br>(3)
22	Foot<br>(Churchill Livingstone; England; English)	4	Not	-	-	-	1 / - / 10-12<br>(4)
23	Medicine (United States)<br>(Lippincott Williams & Wilkins; United States; English)	52	Yes	12,290<br>(99.77%)	1.4	1.3	1, 2 / 5 / 10-12, 15<br>(7)
24	Diabetes, Metabolic Syndrome and Obesity<br>(Dove Medical Press LTD; New Zealand; English)	1	Yes	1112<br>(98.74%)	2.8	2.7	1, 2 / 5 / 11, 13-15<br>(7)
25	Diabetologe<br>(Springer Heidelberg; Germany; German)	8	Not	177<br>(1.69%)	0.352<br>(JCR 2021)	0.324<br>(JCR 2021)	1 / - / 11<br>(2)
26	Klinichna khirurhiia / Ministerstvo okhorony zdorov'ia Ukraïny, Naukove tovarystvo khirurhiv Ukraïny<br>(Nauachnoe Oobshchestvo Khirurgov Ukrainy; Ukraine; Ukrainian)	6	Yes	-	-	-	1 / 5 / -<br>(2)
27	International Journal of Diabetes in Developing Countries<br>(Springer India; India; English)	4	Not	370<br>(10%)	0.7	0.7	1, 2/ 7, 8 / 10, 11, 14, 16, 21<br>(9)
28	Cochrane Database of Systematic Reviews<br>(Wiley; England; English)	12	Not	1143<br>(0%)	8.8	8.6	1, 2 / - / 11<br>(3)
29	Clinics in Podiatric Medicine and Surgery<br>(W B Saunders CO-Elsevier INC; United States; English)	4	Not	157<br>(0%)	0.7	0.7	1, 2 / - / 10-12<br>(5)
30	Khirurgiia (Mosk)<br>(Moskva: Media Sfera; Russia; Russian and English)	12	Yes	-	-	-	1 / - / 11, 12<br>(3)
31	Journal of Vascular Surgery<br>(Mosby-Elsevier; United States; English)	12	Not	1243<br>(13.27%)	3.9	3.2	1, 2 / - 11, 12, 15<br>(5)

Rank	Journal<br>(Publisher; Region; Language)	Frequency<br>(issues/year)	Journal OA	Total citable<br>(%OA)	JIF<br>(2023)	JIF without self citations<br>(2023)	*Diffusion<br>C/M/E databases<br>(Num. databases)
32	Current Diabetes Reviews<br>(Bentham Science Publ LTD; U Arab Emirates; English)	9	Not	304<br>(3.95%)	2.4	2.3	1, 4 / - / 11, 12<br>(4)
33	Journal of Diabetes Science and Technology<br>(SAGE Publications INC; United States; English)	4	Not	538<br>(20.26%)	4.1	3.5	1, 4 / - / 11, 12<br>(4)
34	Zhongguo xiu fu chong jian wai ke za zhi = Zhongguo xiufu chongjian waike zazhi = Chinese journal of reparative and reconstructive surgery<br>(Zhongguo Xiufu Chonjian Waike Zazhi Bianjibu; China; Chinese)	12	Yes	-	-	-	1 / - / 12, 13<br>(3)
35	Foot and Ankle International<br>(SAGE Publications INC; United States; English)	12	Not	508<br>(6.89%)	2.4	1.9	1, 2 / - / 10-12, 27<br>(6)
36	Journal of Tissue Viability<br>(Elsevier SCI LTD; England; English)	4	Not	279<br>(20.79%)	2.4	2.0	1-3 / - / 10-12<br>(6)
37	Journal of Diabetes and Metabolic Disorders<br>(Springer Int Publ AG; England; English)	U	Not	644<br>(9.32%)	1.8	1.7	1, 4 / 7, 9 / 11, 14<br>(6)
38	Chinese Journal of Tissue Engineering Research<br>(Publishing House of Chinese Journal of Tissue Engineering Research; China; Chinese and English)	36	Yes	-	-	-	1 / - / -<br>(1)

Abbreviations: OA: open access; Total citable (% OA): the data included summarizes the items published in the journal in the JCR data year and in the 2 previous years. This 3-year set of published items is used to provide descriptive analysis of the content and community of the journal; JIF (journal impact factor); U: uninterrupted [Information from JCR database]; Diffusion C/M/E databases (citation / multidisciplinary / specialized databases); Num.: number of databases in which the journals are indexed. [Information from MIAR database].

Bibliographic databases in the "*Diffusion" column:

A) Citation databases

1. Scopus (Elsevier); 2. Science Citation Index Expanded (Clarivate); 3. Social Sciences Citation Index (Clarivate); 4. Emerging Sources Citation Index (Clarivate).

B) Multidisciplinary databases

5. DOAJ; 6. Dialnet (University of La Rioja), 7. Academic Search Ultimate (EBSCO); 8. Natural Science Collection (ProQuest); 9. Middle East & Africa Database (ProQuest).

C) Specialized databases

10. CINAHL (EBSCO); 11. EMBASE (Elsevier); 12. Medline (USA, National Library of Medicine); 13. Biomedical Reference Collection: Corporate Edition (EBSCO); 14. Health Research Premium Collection (ProQuest);15. BIOSIS; 16. Pharma Collection (ProQuest); 17. Chemical Abstract Core (American Chemical Society); 18. Index Islamicus (Brill); 19. Public Affairs Index (EBSCO); 20. Advance Technologies & Aeroespace Database (ProQuest); 21. Biological Science Database (ProQuest); 22. Linguistic Bibliography (Brill); 23. Material Science & Engineering Collection (ProQuest); 24. Technology Collection (ProQuest); 25. PsycInfo (American Phychological Association- APA); 26. zbMATH; 27. SPORTDiscus with Full Text (EBSCO).
