# Supplementary material for: Global Trends in Diabetic Foot Research (2004–2023): A Bibliometric Study Based on the Scopus Database
Source: Int J Environ Res Public Health. 2025 Mar 21;22(4):463. doi: 10.3390/ijerph22040463 (PMC12026514; doi:10.3390/ijerph22040463)
Supplement: Supplementary file 1 [file ijerph-22-00463-s001.zip › NewTableS2-Suppl.Mat_ijerph-3461218.pdf]

Table S2: Distribution of the languages of publication of documents on diabetic foot in the period 2004-2023.

Rank	Languages	Num. Documents	Percentage
**1**	English	5890	82.54
**2**	Chinese	369	5.17
**3**	German	183	2.56
**4**	Russian	179	2.51
**5**	Spanish	144	2.02
**6**	French	94	1.32
**7**	Czech	41	0.57
**8**	Ukrainian	41	0.57
**9**	Japanese	41	0.57
**10**	Turkish	26	0.36
**11**	Portuguese	24	0.34
**12**	Persian	22	0.31
**13**	Italian	15	0.21
**14**	Polish	12	0.17
**15**	Korean	9	0.13
**16**	Slovak	9	0.13
**17**	Croatian	7	0.10
**18**	Romanian	5	0.07
**19**	Hungarian	4	0.06
**20**	Dutch	4	0.06
**21**	Greek	4	0.06
**22**	Hebrew	4	0.06
**23**	Arabic	2	0.03
**24**	Bosnian	2	0.03
**25**	Bulgarian	2	0.03
**26**	Finnish	1	0.01
**27**	Danish	1	0.01
**28**	Azerbaijani	1	0.01
	**Total:**	**7136**	**100.00**
