# Supplementary material for: Global Trends in Diabetic Foot Research (2004–2023): A Bibliometric Study Based on the Scopus Database
Source: Int J Environ Res Public Health. 2025 Mar 21;22(4):463. doi: 10.3390/ijerph22040463 (PMC12026514; doi:10.3390/ijerph22040463)
Supplement: Supplementary file 1 [file ijerph-22-00463-s001.zip › NewTableS3-Suppl.Mat_ijerph-3461218.pdf]

Table S3: Affiliations of authors with 20 or more papers per order of production.

Rank	Author (ID Scopus)	TPs	PF	Affiliation more recent in Scopus	City	Country	Type of affiliation
1	Armstrong. David G. (7404407396)	160	31.69	Keck School of Medicine of USC	Los Angeles	United States	Educational institution
2	Lipsky. Benjamin A. (7006768971)	108	25.61	Green Templeton College	Oxford	United Kingdom	Educational institution
3	Lázaro-Martínez. José Luis (18434633300)	86	14.99	Universidad Complutense de Madrid	Madrid	Spain	Educational institution
4	Lavery. Lawrence A. (7006066609)	76	14.17	UT Southwestern Medical School	Dallas	United States	Educational institution
5	Boulton. Andrew J.M. 7202295225)	61	21.55	University of Miami	Coral Gables	United States	Educational institution
6	Edmonds. Michael (16439677500)	57	17.02	King's College Hospital	London	United Kingdom	Health Center
7	Piaggesi. Alberto (7004496777)	51	7.57	Monash University	Melbourne	Australia	Educational institution
8	Morbach. Stephan (6603754743)	49	10.5	Marien Krankenhaus. Soest	Soest	Germany	Health Center
9	Aragón-Sánchez. Javier (6507768519)	47	14.46	La Paloma Hospital	Las Palmas de Gran Canaria	Spain	Health Center
10	Ran. Xingwu (35269932400)	45	8.7	West China School of Medicine/West China Hospital of Sichuan University	Chengdu	China	Educational institution /Health Center
11	Papanas. Nikolaos (12763313600)	44	11.68	Democritus University of Thrace	Komotini	Greece	Educational institution
12	Jude. Edward B. (7003521236)	43	6.95	Tameside and Glossop Integrated Care NHS Foundation Trust	Ashton-under-Lyne	United Kingdom	Health Center
13	Lobmann. Ralf (6701744779)	43	12.54	Klinikum Stuttgart	Stuttgart	Germany	Health Center
14	Uccioli. Luigi (56015453000)	43	5.99	CTO Andrea Alesini	Rome	Italy	Health Center
15	Uçkay. Ilker (22935926900)	42	8.21	Uniklinik Balgrist	Zurich	Switzerland	Health Center
16	García-Álvarez. Yolanda (36236825300)	39	6.53	Universidad Complutense de Madrid	Madrid	Spain	Educational institution

Rank	Author (ID Scopus)	TPs	PF	Affiliation more recent in Scopus	City	Country	Type of affiliation
17	Jirkovská. Alexandra (57197802703)	38	6.13	Institutu Klinické a Experimentální Medicíny	Prague	Czech Republic	Health Center
18	Rümenapf. Gerhard (7004032315)	38	8.07	Deaconess Foundation Hospital	Speyer	Germany	Health Center
19	Apelqvist. Jan (7004430070)	37	7.22	Skånes Universitetssjukhus	Lund	Sweden	Health Center
20	García-Morales. Esther (18434169300)	37	6.28	Universidad Complutense de Madrid	Madrid	Spain	Educational institution
21	Lavigne. Jean-Philippe (24531297400)	37	6.12	Université de Montpellier	Montpellier	France	Educational institution
22	Álvaro-Afonso. Francisco Javier (55628422700)	36	6.23	Universidad Complutense de Madrid	Madrid	Spain	Educational institution
23	Bus. Sicco A. (7004095333)	34	9.83	Universiteit van Amsterdam	Amsterdam	Netherlands	Educational institution
24	Spraul. Maximilian (7004926811)	34	6.35	Diabetes Schwerpunktpraxis	Rheine	Germany	Health Center
25	van Netten. Jaap J. (23983869500)	33	6.84	Universiteit van Amsterdam	Amsterdam	Netherlands	Educational institution
26	Sotto. Albert (7006249763)	32	5.29	Centre Hospitalier Universitaire de Nîmes	Nimes	France	Health Center
27	Meloni. Marco (57215300329)	31	4.03	Università degli Studi di Roma "Tor Vergata"	Rome	Italy	Educational institution
28	Reike. Heinrich (6602994983)	31	4.79	Mariannen-Hospital Werl	Werl	Germany	Health Center
29	Schaper. Nicolaas C. (7004156278)	31	5.59	Universiteit Maastricht	Maastricht	Netherlands	Educational institution
30	Wukich. Dane K. (6602215301)	31	7.66	UT Southwestern Medical Center	Dallas	United States	Health Center
31	Fejfarová. Vladimíra (6602334784)	30	4.35	Institutu Klinické a Experimentální Medicíny	Prague	Czech Republic	Health Center
32	Frykberg. Robert G. (7003666198)	30	8.61	Fountain Hills	Fountain Hills	United States	Health Center
33	Kirsner. Robert S. (7101773124)	29	6.79	University of Miami Leonard M. Miller School of Medicine	Miami	United States	Educational institution
34	Iacopi. Elisabetta (50161569500)	28	4.42	Università di Pisa	Pisa	Italy	Educational institution

Rank	Author<br>(ID Scopus)	TPs	PF	Affiliation more recent in Scopus	City	Country	Type of affiliation
35	Giurato. Laura<br>(35933127700)	27	4.24	Università degli Studi di Roma "Tor Vergata"	Rome	Italy	Educational institution
36	van Baal. Jeff G.<br>(7004075327)	27	4.29	Ziekenhuisgroep Twente	Almelo	Netherlands	Health Center
37	Bém. Robert<br>(12139074100)	26	5.04	Institutu Klinické a Experimentální Medicíny	Prague	Czech Republic	Health Center
38	Larijani. Bagher<br>(57205307740)	26	4.74	Endocrinology and Metabolism Research Institute (TUMS)	Tehran	Iran	Other
39	Lazzarini. Peter A.<br>(41561537500)	26	4.71	Queensland University of Technology	Brisbane	Australia	Educational institution
40	Peters. Edgar J.G.<br>(57217629913)	26	5.78	Amsterdam UMC - University of Amsterdam	Amsterdam	Netherlands	Educational institution
41	Veves. Aristidis<br>(35595507100)	25	4.81	Harvard Medical School	Boston	United States	Educational institution
42	Zgonis. Thomas<br>(6701546705)	25	8.83	The University of Texas Health Science Center at San Antonio	San Antonio	United States	Educational institution
43	Bowling. Frank L.<br>(14062925300)	24	5.91	Manchester Royal Infirmary	Manchester	United Kingdom	Health Center
44	Dubský. Michal<br>(56422302100)	24	2.97	Institutu Klinické a Experimentální Medicíny	Prague	Czech Republic	Health Center
45	Game. Frances<br>(6701759105)	24	7.52	University Hospitals of Derby and Burton NHS Foundation Trust	Derby	United Kingdom	Health Center
46	Jeffcoate. William J.<br>(57203073839)	24	5.16	Nottingham University Hospitals NHS Trust	Nottingham	United Kingdom	Health Center
47	La Fontaine. Javier<br>(24168754900)	24	3.87	The University of Texas Rio Grande Valley	Brownsville	United States	Educational institution
48	Richard. J.-L.<br>(55608085700)	24	6.03	Centre Hospitalier Universitaire de Nîmes	Nimes	France	Health Center
49	Risse. Alexander<br>(55887966800)	24	4.69	Diabeteszentrum am Sophie-Charlotte-Platz	Berlin	Germany	Health Center
50	Senneville. Eric<br>(7003355556)	24	7.78	Gustave Dron Hospital	Tourcoing	France	Health Center
51	Tardáguila-García. Aroa<br>(57193884183)	24	4.08	Universidad Complutense de Madrid	Madrid	Spain	Educational institution
52	Malone. Matthew<br>(55928381000)	23	3.79	Liverpool Hospital	Liverpool	Australia	Health Center

Rank	Author (ID Scopus)	TPs	PF	Affiliation more recent in Scopus	City	Country	Type of affiliation
53	Mohajeri-Tehrani. Mohammad Reza (15728245200)	23	3.31	Endocrinology and Metabolism Research Institute (TUMS)	Tehran	Iran	Other
54	Carter. Marissa J. (16834696100)	22	3.78	Strategic Solutions	Bozeman	United States	Other
55	Chen. Dawei (57148912500)	22	3.07	West China School of Medicine/West China Hospital of Sichuan University	Chengdu	China	Educational institution /Health Center
56	Goretti. Chiara (54883016100)	22	3.74	Università di Pisa	Pisa	Italy	Educational institution
57	López-Moral. Mateo (57210151169)	22	3.67	Universidad Complutense de Madrid	Madrid	Spain	Educational institution
58	Sanz-Corbalán. Irene (55556236600)	22	3.7	Universidad Complutense de Madrid	Madrid	Spain	Educational institution
59	Viswanathan. Vijay (55767770100)	22	5.8	M.V. Hospital for Diabetes	Chennai	India	Health Center
60	Wang. Chun (55860799369)	22	2.98	West China School of Medicine/West China Hospital of Sichuan University	Chengdu	China	Educational institution /Health Center
61	Xu. Zhangrong (55648766000)	22	7.11	PLA Strategic Support Force Characteristic Medical Center	Beijing	China	Health Center
62	Han. Seung-Kyu (26040694200)	21	4.8	Korea University Guro Hospital	Seoul	South Korea	Educational institution /Health Center
63	Hartemann. A. (36966232500)	21	6.07	Hôpital Universitaire Pitié Salpêtrière	Paris	France	Health Center
64	Molines-Barroso. Raúl J. (55773421900)	21	3.5	Universidad Complutense de Madrid	Madrid	Spain	Educational institution
65	Van Acker. Kristien (7006331968)	21	2.88	Centre de Santé des Fagnes Clinique Chimay	Chimay	Belgium	Health Center
66	Ahmad. Jamal (7102588381)	20	6.9	Aligarh Muslim University	Aligarh	India	Educational institution
67	Galiano. Robert D. (6603936643)	20	2.65	Northwestern University Feinberg School of Medicine	Chicago	United States	Educational institution
68	Mauricio. Didac (7004517251)	20	1.71	Universitat de Vic - Universitat Central de Catalunya (UVic-UCC)	Vic	Spain	Educational institution
69	Mills. Joseph L. (7402862404)	20	5.51	Baylor College of Medicine	Houston	United States	Educational institution
70	Müller. Eckhard (8124559900)	20	3.77	KfH-Nierenzentrum	Bernkastel-Kues	Germany	Health Center

Rank	Author<br>(ID Scopus)	TPs	PF	Affiliation more recent in Scopus	City	Country	Type of affiliation
71	Serena. Thomas E.<br>(8878304500)	20	3.21	SerenaGroup Research Foundation	Cambridge	United States	Other
72	Wu. Stephanie C.<br>(9632686300)	20	5.4	Rosalind Franklin University of Medicine and Science	North Chicago	United States	Educational institution
73	Zelen. Charles M.<br>(6506228440)	20	2.96	Professional Education and Research Institute	Roanoke	United States	Other

Abbreviations: TPs: total publications; PF: publications fractionalised.
