# Supplementary material for: Global Trends in Diabetic Foot Research (2004–2023): A Bibliometric Study Based on the Scopus Database
Source: Int J Environ Res Public Health. 2025 Mar 21;22(4):463. doi: 10.3390/ijerph22040463 (PMC12026514; doi:10.3390/ijerph22040463)
Supplement: Supplementary file 1 [file ijerph-22-00463-s001.zip › NewTableS4-Suppl.Mat_ijerph-3461218.pdf]

Table S4: Cluster distribution of the 139 authors that make up the co-authorship network.

N	Cluster (N authors)	Author	Total link strength	Documents in cluster	Citations
1	1 (25 authors)	Armstrong, David G.	198	138	10,266
2		Lavery, Lawrence A.	159	70	5444
3		Boulton, Andrew J.M.	81	55	8266
4		Frykberg, Robert G.	22	26	818
5		Kirsner, Robert S.	28	26	1918
6		Wukich, Dane K.	36	26	937
7		Bowling, Frank L.	28	23	732
8		La Fontaine, Javier	60	22	678
9		Malone, Matthew	26	21	521
10		Peters, Edgar J.G.	49	20	1731
11		Wu, Stephanie C.	29	20	795
12		Lantis, John C.	10	15	541
13		Kim, Paul J.	35	14	553
14		Rajbhandari, Satyan	9	13	407
15		Attinger, Christopher E.	28	12	559
16		Vileikyte, Loretta	19	12	2865
17		Bhavan, Kavita	37	11	327
18		Crews, Ryan T.	17	11	344
19		Margolis, David J.	14	11	768
20		Oz, Orhan K.	31	11	238
21		Raspovic, Katherine Marie	18	11	386
22		Reeves, Neil D.	15	11	512
23		Steinberg, John S.	19	10	225
24		Van Asten, Suzanne A.V.	32	10	403
25		Vickery, Karen	10	10	403
26	2 (15 authors)	Papanas, Nikolaos	43	42	870
27		Edmonds, Michael	27	35	1473
28		Jude, Edward B.	44	26	745
29		Viswanathan, Vijay	7	20	591
30		Maltezos, Efstratios	20	17	528
31		Tentolouris, Nikolaos	11	15	468
32		Abbas, Zulfiqarali G.	10	14	542
33		Dhamodharan, Umapathy	11	13	312
34		Vas, Prashanth	14	13	304
35		Chockalingam, Nachiappan	7	11	185
36		Driver, Vickie R.	7	11	975
37		Formosa, Cynthia	4	11	144
38		Ahluwalia, Raju	19	10	206
39		Kesavan, Rajesh	12	10	325
40		Rastogi, Ashu	5	10	235
41	3 (15 authors)	Lavigne, Jean-Philippe	76	34	1076
42		Sotto, Albert	72	29	939
43		Richard, Jean-Louis	44	22	739
44		Jeffcoate, William J.	34	21	1759
45		Game, Frances	24	19	841
46		Senneville, Eric	15	18	1138
47		Hartemann, Agnès	6	17	571
48		Mills, Joseph L.	29	16	958

N	Cluster (N authors)	Author	Total link strength	Documents in cluster	Citations
49		Dunyach-Remy, Catherine	35	15	306
50		Schuldiner, Sophie	33	13	382
51		Boyko, Edward J.	18	12	1593
52		Jourdan, Nathalie	34	12	525
53		Monteiro-Soares, Matilde	18	10	898
54	4 (13 authors)	Bus, Sicco A.	40	32	4544
55		Van Netten, Jaap J.	48	30	1490
56		Fejfarová, Vladimíra	63	25	416
57		Jirkovská, Alexandra	60	23	395
58		Lazzarini, Peter A.	12	22	616
59		Apelqvist, Jan	21	21	3673
60		Bém, Robert	58	21	350
61		Dubský, Michal	57	19	363
62		Van Baal, Jeff G.	22	19	478
63		Schaper, Nicolaas C.	28	18	2057
64		Chadwick, Paul	11	16	370
65		Mrozikiewicz-Rakowska, Beata	2	12	105
66		Hinchliffe, Robert J.	13	10	945
67	5 (13 authors)	Lázaro-Martínez, José Luis	269	76	1524
68		Aragón-Sánchez, Javier	95	46	1538
69		García-Álvarez, Yolanda	169	39	384
70		García-Morales, Esther	144	36	922
71		Álvaro-Afonso, Francisco Javier	157	36	441
72		Tardáguila-García, Aroa	106	24	176
73		López-Moral, Mateo	96	22	73
74		Sanz-Corbalán, Irene	98	22	221
75		Molines-Barroso, Raúl J.	100	21	335
76		García-Madrid, Marta	78	18	68
77		Beneit-Montesinos, Juan Vicente	37	12	544
78		Víquez-Molina, Gerardo	23	12	71
79		López-Valverde, María Eugenia	21	10	70
80	6 (12 authors)	Piaggesi, Alberto	69	36	648
81		Uccioli, Luigi	62	26	726
82		Iacopi, Elisabetta	58	25	335
83		Giurato, Laura	56	22	562
84		Goretti, Chiara	55	22	250
85		Meloni, Marco	54	22	343
86		Coppelli, Alberto	38	13	201
87		Izzo, Valentina	37	11	223
88		Tedeschi, Anna	20	11	404
89		Anichini, Roberto	14	10	261
90		Brocco, Enrico	18	10	180
91		Seghieri, Giuseppe	9	10	245
92	7 (11 authors)	Morbach, Stephan	158	46	660
93		Lobmann, Ralf	71	37	860
94		Rümenapf, Gerhard	127	36	225
95		Risse, Alexander	115	23	120
96		Spraul, Maximilian	111	23	112
97		Reike, Heinrich	112	21	114
98		Müller, Eckhard	111	20	111
99		Engels, Gerald	18	13	83
100		Hochlenert, D.	16	12	66

N	Cluster (N authors)	Author	Total link strength	Documents in cluster	Citations
101		Lawall, Holger	6	12	144
102		Eckhard, Michael	65	11	26
103	8 (10 authors)	Lipsky, Benjamin A.	160	98	6715
104		Uçkay, Ilker	87	40	995
105		Gariani, Karim	37	15	380
106		Waibel, Felix W. A.	41	14	85
107		Uzun, Günalp	14	13	381
108		Mutluoglu, Mesut	15	12	317
109		Schöni, Madlaina	35	12	62
110		Ertugrul, Bulent M.	7	11	199
111		Kressmann, Benjamin	31	11	254
112		Berli, Martin C.	29	10	94
113	9 (9 authors)	Carter, Marissa J.	95	21	412
114		Didomenico, Lawrence A.	70	13	338
115		Galiano, Robert D.	92	19	386
116		Glat, Paul M.	60	11	99
117		Orgill, Dennis P.	90	17	322
118		Reyzelman, Alexander M.	27	15	602
119		Serena, Thomas E.	50	17	668
120		Snyder, Robert J.	20	13	462
121		Zelen, Charles M.	94	19	653
122	10 (8 authors)	Ran, Xingwu	51	39	243
123		Chen, Dawei	38	19	157
124		Wang, Chun	40	19	155
125		Xu, Zhangrong	13	17	188
126		Wang, Aihong	7	11	12
127		Yang, Chuan	1	11	90
128		Chen, Lihong	23	10	117
129		Chen, Mingwei	1	10	38
130	11 (3 authors)	Abularrage, Christopher J.	21	13	642
131		Hicks, Caitlin W.	21	12	731
132		Mathioudakis, Nestoras	20	11	1018
133	12 (3 authors)	Huang, Yu-Yao	23	14	331
134		Huang, Chung-Huei	20	10	274
135		Lin, Cheng-Wei	20	10	147
136	13 (2 authors)	Veves, Aristidis	1	15	868
137		Schmidt, Brian M.	3	13	109
138	14 (2 authors)	Zgonis, Thomas	16	23	350
139		Ramanujam, Crystal L.	12	12	105

Note: Authors with 10 or more published documents and a maximum of 10 signatures per article in the period 2004-2023 sorted by highest to lowest scientific production on diabetic foot. Data exported from the co-authoring network generated with VOSviewer.
