# Supplementary material for: Global Trends in Diabetic Foot Research (2004–2023): A Bibliometric Study Based on the Scopus Database
Source: Int J Environ Res Public Health. 2025 Mar 21;22(4):463. doi: 10.3390/ijerph22040463 (PMC12026514; doi:10.3390/ijerph22040463)
Supplement: Supplementary file 1 [file ijerph-22-00463-s001.zip › NewTableS5-Suppl.Mat_ijerph-3461218.pdf]

Table S5: Journals conforming the core of knowledge on diabetic foot according to Bradford's areas.

Rank	Journal (ISSN)	TPs	% (7136)	H-Index (2023)	SJR (2023)	Quartile (2023)	Category
1	International Journal of Lower Extremity Wounds (15347346)	278	15.37	49	0.43	Q2	Surgery
2	International Wound Journal (17424801, 1742481X)	210	11.61	83	0.73	Q1	Dermatology
3	Journal of Wound Care (09690700, 20522916)	158	8.73	77	0.4	Q2	Fundamentals and Skills
4	Wounds (10447946, 19432704)	96	5.31	49	0.3	Q2	Medical and Surgical Nursing
5	Diabetes Research and Clinical Practice (01688227, 18728227)	96	5.31	140	1.34	Q1	Endocrinology
6	Diabetes/Metabolism Research and Reviews (15207552, 15207560)	94	5.20	135	1.99	Q1	Endocrinology
7	Journal of the American Podiatric Medical Association (19308264, 87507315)	85	4.70	65	0.2	Q3	Podiatry
8	Wound Repair and Regeneration (10671927, 1524475X)	84	4.64	133	0.8	Q1	Dermatology
9	Diabetes Care (01495992, 19355548)	79	4.37	418	5.69	Q1	Advanced and Specialized Nursing
10	Diabetic Medicine (07423071, 14645491)	72	3.98	165	1.3	Q1	Endocrinology
11	Advances in Skin and Wound Care (15277941, 15388654)	62	3.43	71	0.4	Q2	Advanced and Specialized Nursing
12	Chinese Journal of Diabetes Mellitus (16745809)	61	3.37	8	0.13	Q4	Endocrinology, Diabetes and Metabolism

Rank	Journal (ISSN)	TPs	% (7136)	H-Index (2023)	SJR (2023)	Quartile (2023)	Category
13	Journal of Clinical Medicine (20770383)	60	3.32	113	0.88	Q1	Medicine (miscellaneous)
14	Diabetes and Metabolic Syndrome: Clinical Research and Reviews (18714021, 18780334)	59	3.26	83	1.31	Q1	Endocrinology, Diabetes and Metabolism
15	Journal of Diabetes and its Complications (10568727, 1873460X)	58	3.21	98	1.02	Q1	Internal Medicine
16	PLoS ONE (19326203)	55	3.04	435	0.84	Q1	Multidisciplinary
17	Journal of Foot and Ankle Surgery (10672516, 15422224)	54	2.99	77	0.7	Q1	Surgery
18	Journal of Diabetes Research (23146745, 23146753)	51	2.82	83	0.99	Q2	Endocrinology
19	Journal of Foot and Ankle Research (17571146)	47	2.60	54	0.7	Q2	Orthopedics and Sports Medicine
20	Frontiers in Endocrinology (16642392)	44	2.43	120	1.24	Q1	Endocrinology, Diabetes and Metabolism
21	Diabetic Foot and Ankle (2000625X)	41	2.27	26	0	-	Podiatry / Internal Medicine
22	Foot 09582592, 15322963	40	2.21	44	0.36	Q2	Podiatry
23	Medicine (United States) (00257974, 15365964)	36	1.99	174	0.44	Q3	Medicine (miscellaneous)
24	Diabetes, Metabolic Syndrome and Obesity (11787007)	35	1.93	64	0.84	Q2	Internal Medicine
25	Diabetologe (18609716, 18609724)	33	1.82	0	0	-	Endocrinology, Diabetes and Metabolism
26	Klinichna khirurhiia / Ministerstvo okhorony zdorov'ia Ukraïny, Naukove tovarystvo khirurhiv Ukraïny (00232130)	32	1.77	6	0	-	Medicine (miscellaneous) / Surgery

Rank	Journal (ISSN)	TPs	% (7136)	H-Index (2023)	SJR (2023)	Quartile (2023)	Category
27	International Journal of Diabetes in Developing Countries (09733930)	32	1.77	33	0.25	Q3	Internal Medicine
28	Cochrane Database of Systematic Reviews (14651858)	32	1.77	327	1.41	Q1	Medicine (miscellaneous)
29	Clinics in Podiatric Medicine and Surgery (08918422, 15582302)	31	1.71	40	0.27	Q3	Orthopedics and Sports Medicine
30	Khirurgiia (Mosk) (00231207, 23095628)	30	1.66	13	0.22	Q3	Surgery
31	Journal of Vascular Surgery (07415214, 10976809)	29	1.60	218	1.94	Q1	Cardiology and Cardiovascular Medicine
32	Current Diabetes Reviews (15733998, 18756417)	29	1.60	68	0.77	Q2	Endocrinology
33	Journal of Diabetes Science and Technology (19322968)	28	1.55	93	1.05	Q1	Bioengineering
34	Zhongguo xiu fu chong jian wai ke za zhi = Zhongguo xiufu chongjian waike zazhi = Chinese journal of reparative and reconstructive surgery (10021892)	27	1.49	16	0.17	Q4	Medicine (miscellaneous)
35	Foot and Ankle International (10711007)	27	1.49	128	1.5	Q1	Orthopedics and Sports Medicine
36	Journal of Tissue Viability (0965206X, 18764746)	26	1.44	41	0.61	Q2	Dermatology
37	Journal of Diabetes and Metabolic Disorders (22516581)	26	1.44	46	0.6	Q2	Internal Medicine
38	Chinese Journal of Tissue Engineering Research (16738225)	26	1.44	12	0.12	Q4	Biomedical Engineering

Abbreviations: TPs: total publications; category: category ranked in the best quartile according to the Scimago and Journal and Country Rank (SJR) in 2023; data for h-index, SJR and quartile are from SJR in 2023.
