# Supplementary material for: Global Trends in Diabetic Foot Research (2004–2023): A Bibliometric Study Based on the Scopus Database
Source: Int J Environ Res Public Health. 2025 Mar 21;22(4):463. doi: 10.3390/ijerph22040463 (PMC12026514; doi:10.3390/ijerph22040463)
Supplement: Supplementary file 1 [file ijerph-22-00463-s001.zip › NewTableS6-Suppl.Mat_ijerph-3461218.pdf]

Table S6: Main editorial characteristics, access characteristics, JCR impact indicators (2023), and dissemination in databases of the journals that form the core of knowledge on diabetic foot according to Bradford's areas.

Rank	Journal (Publisher; Region; Language)	Frequency (issues/year)	Journal OA	Total citable (%OA)	JIF (2023)	JIF without self citations (2023)	*Diffusion C/M/E databases (Num. databases)
1	International Journal of Lower Extremity Wounds (SAGE Publications Inc.; United States; English)	4	Not	341 (2.64%)	1.5	1.3	1, 2 / 6 / 10-12 (6)
2	International Wound Journal (Wiley; England; English)	6	Yes	899 (81.87%)	2.6	2.3	1, 2 / 5 / 10-14 (8)
3	Journal of Wound Care (MA Healthcare LTD; England; English)	12	Not	500 (0%)	1.5	1.3	1, 2 / - / 10-14 (7)
4	Wounds (HMP Communications; United States; English)	12	Not	211 (0%)	1.4	1.2	1, 2 / - / 11, 12 (4)
5	Diabetes Research and Clinical Practice (Elsevier Ireland LTD; Netherlands; English)	12	Not	1136 (23.50%)	6.1	5.9	1, 2 / 7 / 10-12, 15, 18 (8)
6	Diabetes/Metabolism Research and Reviews (Wiley; England; English)	8	Not	272 (39.71%)	4.6	4.3	1, 2 / - / 10-15 (8)
7	Journal of the American Podiatric Medical Association (American Podiatric Medical Association; United States; English)	6	Not	349 (0%)	0.5	0.5	1, 2 / - / 10-13 (4)
8	Wound Repair and Regeneration (Wiley; United States; English)	6	Not	210 (24.76%)	3.8	3.8	1, 2 / 7 / 10-13, 15 (8)
9	Diabetes Care (American Diabetes Association; Unites States; English)	12	Not	996 (1.2%)	14.8	14.2	1, 2 / 6-8 / 10-14, 16, 19 (12)
10	Diabetic Medicine (Wiley; England; English)	12	Not	578 (43.08%)	3.2	3.0	1, 2 / 7 / 10-14 (8)
11	Advances in Skin and Wound Care (Lippincott Williams & Wilkins; United States; English)	12	Not	319 (8.78%)	1.7	1.6	1-3 / - / 10-12, 14 (7)
12	Chinese Journal of Diabetes Mellitus (Chinese Medical Journals Publishing House Co.Ltd; China; Chinese)	12	Not	-	-	-	1 / - / - (1)
13	Journal of Clinical Medicine (MDPI; Switzerland; English)	24	Yes	20,573 (99.73%)	3.0	2.8	1, 2 / 7 / 11, 14 (5)
14	Diabetes and Metabolic Syndrome: Clinical Research and Reviews (Elsevier; India; English)	6	Not	651 (4.61%)	4.3	4.3	1, 4 / - / 11, 12, 17 (5)

Rank	Journal<br>(Publisher; Region; Language)	Frequency<br>(issues/year)	Journal OA	Total citable<br>(%OA)	JIF<br>(2023)	JIF without self citations<br>(2023)	*Diffusion<br>C/M/E databases<br>(Num. databases)
15	Journal of Diabetes and its Complications<br>(Elsevier SCI LTD; United States; English)	6	Not	475<br>(15.79%)	2.9	2.8	1, 2 / 7 / 10-12, 14-16<br>(9)
16	PLoS ONE<br>(Public Library Science; United States; English)	U	Yes	46,172<br>(99.34%)	2.9	2.8	1, 2/ 5, 7, 8 / 11-18, 20-26<br>(20)
17	Journal of Foot and Ankle Surgery<br>(Elsevier Science INC; United States; English)	6	Not	663<br>(5.43%)	1.3	1.2	1, 2 / - / 10-12<br>(5)
18	Journal of Diabetes Research<br>(Hindawi LTD; United States; English)	12	Yes	405<br>(99.75%)	3.6	3.6	1, 2 / 5, 7 / 11-14<br>(8)
19	Journal of Foot and Ankle Research<br>(BMC; England; English)	1	Yes	244<br>(100%)	2.5	2.2	1, 2 / 5, 7 / 11, 12, 14<br>(7)
20	Frontiers in Endocrinology<br>(Frontiers Media SA; Switzerland; English)	1	Yes	8233<br>(99.7%)	3.9	3.6	1, 2 / 5 / 11, 12, 15<br>(6)
21	Diabetic Foot and Ankle<br>(Taylor & Francis LTD; England; English)	1	Yes	8<br>(87.50%)	-	-	1, 4 / - / 14<br>(3)
22	Foot<br>(Churchill Livingstone; England; English)	4	Not	-	-	-	1 / - / 10-12<br>(4)
23	Medicine (United States)<br>(Lippincott Williams & Wilkins; United States; English)	52	Yes	12,290<br>(99.77%)	1.4	1.3	1, 2 / 5 / 10-12, 15<br>(7)
24	Diabetes, Metabolic Syndrome and Obesity<br>(Dove Medical Press LTD; New Zealand; English)	1	Yes	1112<br>(98.74%)	2.8	2.7	1, 2 / 5 / 11, 13-15<br>(7)
25	Diabetologe<br>(Springer Heidelberg; Germany; German)	8	Not	177<br>(1.69%)	0.352<br>(JCR 2021)	0.324<br>(JCR 2021)	1 / - / 11<br>(2)
26	Klinichna khirurhiia / Ministerstvo okhorony zdorov'ia Ukraïny, Naukove tovarystvo khirurhiv Ukraïny<br>(Nauachnoe Oobshchestvo Khirurgov Ukrainy; Ukraine; Ukrainian)	6	Yes	-	-	-	1 / 5 / -<br>(2)
27	International Journal of Diabetes in Developing Countries<br>(Springer India; India; English)	4	Not	370<br>(10%)	0.7	0.7	1, 2/ 7, 8 / 10, 11, 14, 16, 21<br>(9)
28	Cochrane Database of Systematic Reviews<br>(Wiley; England; English)	12	Not	1143<br>(0%)	8.8	8.6	1, 2 / - / 11<br>(3)
29	Clinics in Podiatric Medicine and Surgery<br>(W B Saunders CO-Elsevier INC; United States; English)	4	Not	157<br>(0%)	0.7	0.7	1, 2 / - / 10-12<br>(5)
30	Khirurgiia (Mosk)<br>(Moskva: Media Sfera; Russia; Russian and English)	12	Yes	-	-	-	1 / - / 11, 12<br>(3)
31	Journal of Vascular Surgery<br>(Mosby-Elsevier; United States; English)	12	Not	1243<br>(13.27%)	3.9	3.2	1, 2 / - 11, 12, 15<br>(5)

Rank	Journal (Publisher; Region; Language)	Frequency (issues/year)	Journal OA	Total citable (%OA)	JIF (2023)	JIF without self citations (2023)	*Diffusion C/M/E databases (Num. databases)
32	Current Diabetes Reviews (Bentham Science Publ LTD; U Arab Emirates; English)	9	Not	304 (3.95%)	2.4	2.3	1, 4 / - / 11, 12 (4)
33	Journal of Diabetes Science and Technology (SAGE Publications INC; United States; English)	4	Not	538 (20.26%)	4.1	3.5	1, 4 / - / 11, 12 (4)
34	Zhongguo xiu fu chong jian wai ke za zhi = Zhongguo xiufu chongjian waike zazhi = Chinese journal of reparative and reconstructive surgery (Zhongguo Xiufu Chonjian Waike Zazhi Bianjibu; China; Chinese)	12	Yes	-	-	-	1 / - / 12, 13 (3)
35	Foot and Ankle International (SAGE Publications INC; United States; English)	12	Not	508 (6.89%)	2.4	1.9	1, 2 / - / 10-12, 27 (6)
36	Journal of Tissue Viability (Elsevier SCI LTD; England; English)	4	Not	279 (20.79%)	2.4	2.0	1-3 / - / 10-12 (6)
37	Journal of Diabetes and Metabolic Disorders (Springer Int Publ AG; England; English)	U	Not	644 (9.32%)	1.8	1.7	1, 4 / 7, 9 / 11, 14 (6)
38	Chinese Journal of Tissue Engineering Research (Publishing House of Chinese Journal of Tissue Engineering Research; China; Chinese and English)	36	Yes	-	-	-	1 / - / - (1)

Abbreviations: OA: open access; Total citable (% OA): the data included summarizes the items published in the journal in the JCR data year and in the 2 previous years. This 3-year set of published items is used to provide descriptive analysis of the content and community of the journal; JIF (journal impact factor); U: uninterrupted [Information from JCR database]; Diffusion C/M/E databases (citation / multidisciplinary / specialized databases); Num.: number of databases in which the journals are indexed. [Information from MIAR database].

Bibliographic databases in the "*Diffusion" column:

A) Citation databases

1. Scopus (Elsevier); 2. Science Citation Index Expanded (Clarivate); 3. Social Sciences Citation Index (Clarivate); 4. Emerging Sources Citation Index (Clarivate).

B) Multidisciplinary databases

5. DOAJ; 6. Dialnet (University of La Rioja), 7. Academic Search Ultimate (EBSCO); 8. Natural Science Collection (ProQuest); 9. Middle East & Africa Database (ProQuest).

C) Specialized databases

10. CINAHL (EBSCO); 11. EMBASE (Elsevier); 12. Medline (USA, National Library of Medicine); 13. Biomedical Reference Collection: Corporate Edition (EBSCO); 14. Health Research Premium Collection (ProQuest);15. BIOSIS; 16. Pharma Collection (ProQuest); 17. Chemical Abstract Core (American Chemical Society); 18. Index Islamicus (Brill); 19. Public Affairs Index (EBSCO); 20. Advance Technologies & Aeroespace Database (ProQuest); 21. Biological Science Database (ProQuest); 22. Linguistic Bibliography (Brill); 23. Material Science & Engineering Collection (ProQuest); 24. Technology Collection (ProQuest); 25. PsycInfo (American Phychological Association- APA); 26. zbMATH; 27. SPORTDiscus with Full Text (EBSCO).
